# IPNA clinical practice recommendations on care of pediatric patients with pre-existing kidney disease during seasonal outbreak of COVID-19

**DOI:** 10.1007/s00467-024-06565-5

**Published:** 2024-12-29

**Authors:** Khalid A. Alhasan, Rupesh Raina, Olivia Boyer, Jean Koh, Melvin Bonilla-Felix, Sidharth K. Sethi, Yasser S. Amer, Paula Coccia, Mohamad-Hani Temsah, Judith Exantus, Samina A. Khan, Xuhui Zhong, Vera Koch, Ali Duzova, Anil Vasudevan, Mignon McCulloch, Upton Allen, Guido Filler, Giovanni Montini, On behalf of the International Pediatric Nephrology Association, Dieter Haffner, Matko Marlais, William Morello, Jodi M. Smith, Vikas Dharnidharka, Isa F. Ashoor, Peter Trnka, Chanel Prestidge, Arvind Bagga, Pankaj Hari, Alison Ma, Mukta Mantan, Luis Ignacio Rodriguez, Jaime M. Restrepo, Nilzete Liberato Bresolin, Hesham Safouh, Rajendra Bhimma, Bashir Admani, Chris Esezobor

**Affiliations:** 1 Department of Pediatrics, College of Medicine, King Saud University, Riyadh, Saudi Arabia; 2 Organ Transplant Center of Excellence, King Faisal Specialist Hospital & Research Center, Kidney & Pancreas Health Center, Riyadh, Saudi Arabia; 3 Department of Nephrology, Cleveland Clinic Akron General and Akron Children Hospital, Akron, OH USA; 4 Paris Cité University, Pediatric Nephrology, Reference Center for Idiopathic Nephrotic Syndrome in Children and Adults, Imagine Institute, Necker Children’s Hospital, APHP, Paris, France; 5 Department of Paediatric Nephrology, Starship Children’s Hospital, Auckland, New Zealand; 6 Department of Pediatrics, University of Puerto Rico-Medical Sciences Campus, San Juan, Puerto Rico; 7 Pediatric Nephrology, Kidney Institute, Medanta, The Medicity Hospital, Gurgaon, 122001 Haryana India; 8 Clinical Practice Guidelines and Quality Research Unit, Quality Management Department, King Saud University Medical City, Riyadh, Saudi Arabia; 9 Internal Medicine Department, Ribeirão Preto Medical School, University of São Paulo, São Paulo, Brazil; 10 Division of Pediatric Nephrology, Department of Pediatrics, Hospital Italiano de Buenos Aires, Buenos Aires, Argentina; 11 Department of Pediatrics, Faculty of Medicine and Pharmacy, State University of Haïti, State University Hospital of Haïti, Port-Au-Prince, Haiti; 12 Department of Primary Care Medicine, Faculty of Medicine, Universiti Malaya, 50603 Kuala Lumpur, Malaysia; 13 Department of Pediatric Nephrology, Peking University First Hospital, Beijing, China; 14 Children’s Institute Hospital das Clinicas Univ Sao Paulo Medical School, Sao Paulo, Brazil; 15 Division of Pediatric Nephrology, Faculty of Medicine, Hacettepe University, Ankara, Türkiye; 16 Department of Pediatric Nephrology, St. John’s Medical College Hospital, St. John’s Academy of Health Sciences, Bengaluru, India; 17 Red Cross War Memorial Children’s Hospital, University of Cape Town, Cape Town, South Africa; 18 Division of Infectious Diseases and the Transplant and Regenerative Medicine Center, Department of Paediatrics, Hospital for Sick Children, University of Toronto, Toronto, ON Canada; 19 Department of Paediatrics, Children’s Hospital, London Health Science Centre, Western University, 800 Commissioners Road East, London, ON N6A 5W9 Canada; 20 Pediatric Nephrology, Dialysis and Transplant Unit, Fondazione IRCCS Ca’ Granda, Ospedale Maggiore Policlinico, Milan, Italy; 21 Department of Clinical Sciences and Community Health, University of Milano, Milan, Italy

**Keywords:** COVID-19, SARS-COV-2, Children, Kidney disease, Pediatrics, Clinical practice, IPNA, Evidence-based

## Abstract

The coronavirus disease 2019 (COVID-19) pandemic, instigated by the severe acute respiratory syndrome coronavirus 2 (SARS-CoV-2), has profoundly impacted healthcare infrastructures around the globe. While children are usually asymptomatic or have mild symptoms, children with pre-existing kidney conditions require specialized attention. This pivotal report, championed by the International Pediatric Nephrology Association (IPNA), delivers precise and actionable recommendations tailored for pediatric patients with kidney ailments in this pandemic landscape. Central to our findings are rigorous infection control protocols. These are particularly stringent in high-risk zones, emphasizing telehealth’s indispensable role, the significance of curtailing in-person consultations, and the imperative of following rigorous guidelines in regions with heightened COVID-19 prevalence. Additionally, the report delves into vaccination approaches for children with kidney issues, highlighting that the choice of vaccine is often governed by regional accessibility and policy frameworks, rather than a universal preference. A notable observation is the potential correlation between COVID-19 vaccines and specific kidney disorders. However, establishing a direct causal link remains elusive. In summary, our research accentuates the critical need for specialized pediatric kidney care during global health crises and reaffirms the continuous research imperative, especially regarding vaccination ramifications.

**Supplementary Information:**

The online version contains supplementary material available at 10.1007/s00467-024-06565-5.

## Introduction

The coronavirus disease 2019 (COVID-19) pandemic, triggered by the severe acute respiratory syndrome coronavirus 2 (SARS-CoV-2), has led to profound implications for healthcare systems globally. Susceptible populations, including children with pre-existing kidney diseases, were particularly impacted [1–3]. Emerging evidence indicates that, akin to adults, kidney involvement has been reported in pediatric patients with COVID-19 [1]. The emergence of multisystem inflammatory syndrome in children (MIS-C) following SARS-CoV-2 infection highlighted that children are not immune to inflammatory manifestations, with potential for multi-organ involvement and dysfunction, including acute kidney injury (AKI) and relapses of previous kidney diseases [1]. The International Pediatric Nephrology Association (IPNA) acknowledged the necessity to provide targeted recommendations for managing pediatric patients with kidney diseases throughout this public health crisis and beyond. The pandemic has necessitated a shift in medical priorities, impacting routine screenings and diagnoses, and highlighting the pressing need for future medical revolutions to improve healthcare resource management strategies [4].

As we navigate the current stage of the COVID-19 pandemic, significant shifts have occurred, particularly marked by the end of the public health emergency status. This transition signifies a critical move towards treating SARS-CoV-2 as an endemic virus, implying that it will persist in the population at a manageable level without causing widespread disruption. The focus now is on integrating COVID-19 management into routine healthcare systems, with ongoing vaccination efforts, surveillance, and public health messaging tailored to encourage individuals to stay updated with boosters and adopt preventive measures, especially during peak seasons [5]. A study by Thompson et al. (2023) highlights the effectiveness of continued vaccination campaigns in reducing severe outcomes and hospitalizations, underscoring the importance of maintaining vigilance and healthcare readiness in this new phase [6].

As we navigate through the evolving landscape of the COVID-19 pandemic, numerous countries are entering a phase where they are shifting from a crisis mode to a strategy focused on living with SARS-CoV-2 as an endemic virus. This transition underscores the importance of vaccination campaigns, including booster doses, as well as robust surveillance systems to monitor virus spread and detect emerging variants. Emphasizing public health measures such as mask-wearing and physical distancing remains crucial in certain contexts, while discussions around quarantine protocols and border controls may evolve as we adapt to a future where pandemics are a continuing consideration [7].

The concept of quarantine and isolation, once central to pandemic response, is becoming less emphasized in everyday practice. However, these measures remain relevant as strategic tools for future pandemics or localized outbreaks of infectious diseases. The pandemic has underscored the importance of having robust public health infrastructures that can swiftly implement quarantine and isolation when necessary to control the spread of disease. Lessons learned from COVID-19 highlight the need for flexibility, resilience, and adaptability in public health policies, ensuring preparedness for future health emergencies. Planning and preparedness for potential pandemics must consider the role of these interventions, integrating them into broader strategies for disease containment and public health protection [5].

The aim of such clinical practice recommendations is to provide evidence-based guidance to clinicians caring for pediatric patients with kidney diseases, including chronic kidney disease (CKD), nephrotic syndrome (NS), and patients undergoing organ transplantation or receiving immunosuppressive therapy. Suggestions are included for the optimal management of AKI and kidney replacement therapy (KRT) in the context of COVID-19. This guideline also holds significance for patients with co-morbidities and for implementing infection control measures in high-risk areas such as hemodialysis (HD) [2, 8].

The recommendations are structured around five principal PICO (population, intervention, comparison, outcomes) questions, each led by a dedicated task force subgroup with expertise in the relevant area. The PICO questions address issues such as best practices for COVID-19 vaccination, infection control measures, precautions for transplant donors and recipients, management of patients suspected or confirmed to have COVID-19 and on immunosuppressive therapy, and considerations for low-income countries (LIC) [9]. IPNA aims at improving patient outcomes with these recommendations, reducing the spread of SARS-CoV-2 infections in high-risk settings, and guiding the management of pediatric patients with kidney diseases beyond the pandemic. It is our hope that these guidelines will serve as a valuable resource for clinicians worldwide in navigating the unique challenges posed by COVID-19 and provide future guidance in similar infectious disease outbreaks [3].

## Methodology

### Overview of the guideline project

We applied the RIGHT (Reporting Items for practice Guidelines in Health Care) Statement for reporting Clinical Practice Guidelines [10]. Three groups were made: a core leadership group, an external expert group, and a voting panel. The core group included 16 members of IPNA, pediatric nephrologists, epidemiologists, a guideline methodologist, a pediatric infectious diseases expert, a clinical pharmacologist, and a pediatric intensivist. The specific expertise and tasks of the core group members are provided in Supplementary Table S1. The external expert group comprised pediatric infectious disease experts and a patient representative.

The voting panel group involved 19 pediatric nephrologists who were representatives from each IPNA Regional Society, with proficiency in the management of children with pre-existing kidney disease, including CKD, NS, and those undergoing transplantation or immunosuppressive therapy, during the COVID-19 era. Members of the voting group were surveyed using an electronic questionnaire, where they were asked to indicate their level of agreement on a 5-point scale (ranging from strongly disagree to strongly agree) following the Delphi method. For topics that did not reach a 70% consensus level, the core group re-evaluated and modified the recommendations. These revised recommendations were then reviewed by the voting panel again, continuing this process until a consensus level greater than 70% was achieved.

### Developing the PICO questions

We developed five primary PICO (Patient or Population covered, Intervention, Comparator, Outcome) questions as follows [11]: Population: Children and adolescents (> 3 months and < 18 years) with pre-existing kidney disease during periods of significant SARS-CoV-2 circulation; Intervention and Comparators: treatment versus no treatment, other treatment or placebo; Outcomes Addressed: We addressed recommendations for the best practices for COVID-19 vaccination, infection control measures, precautions for transplant donors and recipients, management of patients suspected or confirmed to have COVID-19 and on immunosuppressive therapy, and considerations for less-resourced countries (LRC) in children with pre-existing kidney disease during SARS-CoV-2 infection. The primary objectives were to enhance patient outcomes, decrease the transmission of COVID-19 in high-risk environments, and provide guidance for managing pediatric patients with kidney diseases, including kidney transplant recipients, during SARS-CoV-2 infection (Supplementary Tables S3 and S4).

### Literature search

We searched the PubMed database for publications before 31 March 2023, and we included systematic reviews of randomized controlled trials (RCTs), prospective uncontrolled trials, observational studies, and registry studies on managing COVID-19 in children with pre-existing kidney disease. The search was restricted to human studies in English. Where feasible, meta-analyses of RCTs using risk ratios (RRs) with 95% confidence intervals (CI) were cited from relevant systematic reviews. More details and the summary of the publications used for these international evidence-based clinical practice recommendations (CPRs) are provided in the supplementary material (Supplementary Table S2).

### Grading system

We adhered to the grading system established by the American Academy of Pediatrics [12]. The quality of evidence was graded as high (A), moderate (B), low (C), very low (D), or not applicable (X). The latter pertains to exceptional cases where validation studies are not feasible because the benefits or harms are overwhelmingly clear. This letter was utilized to grade contraindications of therapeutic measures and safety parameters. The strength of each recommendation was classified as strong, moderate, weak, or discretionary (when no recommendation could be made).

## Clinical practice recommendations

1. **COVID-19 vaccination for children with kidney disease, including those who are immunocompromised or have co-morbidities**
2. **Preferred COVID-19 vaccine for children with kidney diseases**

### Recommendation:

No firm recommendation can be made regarding a single preferred vaccine for children with kidney disease across the various jurisdictions internationally.

**Evidence and rationale**

### Categories of available vaccines:

The recommended vaccines are typically influenced by accessibility and governed by jurisdictional policies. There are different types of COVID-19 vaccines in use in various jurisdictions around the world [13–15]. Whole virus (inactivated) vaccines use the entire virus to trigger an immune response, while subunit vaccines use only parts of the virus to trigger the immune system. Viral vector vaccines use a harmless virus to deliver genetic material that provides instructions for making specific proteins. Finally, there are nucleic acid vaccines, which use only the genetic material of the virus to provide instructions for making specific proteins, but not the whole virus.

Each type of vaccine has its advantages and disadvantages, and the type of vaccine used depends on several factors, including accessibility in different jurisdictions around the world. In the above regard, different types of vaccines are being used in various regions around the world.

A list of products that have received full World Health Emergency Use Listing (EUL) that was updated by WHO up to December 2, 2022, is in Table 1 [13]. These products are available in different jurisdictions and their approval status has evolved over time. For example, in the USA, the FDA has reduced the list of approved vaccines to 3 products [16] (as of 26/6/23; Novavax COVID-19 vaccine adjuvanted, Moderna Bivalent [Spikevax] and Pfizer/BioNTech Bivalent (Comirnaty)). In the United Kingdom of Great Britain and Northern Ireland, 8 vaccines have been approved for use [17]. These include 4 mRNA products, 2 non-replicating viral vector vaccines, 1 protein subunit vaccine, and 1 inactivated viral vaccine.

**Table 1:** Vaccines granted emergency use listing (EUL) by WHO

Vaccine [brand name]	Type of product	Number of countries in which product has been approved
Serum Institute of India [COVOVAX (Novavax formulation)]	Protein subunit	6
Novavax [Nuvaxovid]	Protein subunit	40
Moderna [Spikevax]	mRNA	88
Pfizer/BioNTech [Comirnaty]	mRNA	149
CanSino [Convidecia]	Non-replicating viral vector	10
Janssen (Johnson & Johnson) [Jcovden]	Non-replicating viral vector	113
Oxford/AstraZeneca* [Vaxzevria]	Non-replicating viral vector	149*
Serum Institute of India [Covishield (Oxford/ AstraZeneca formulation)]	Non-replicating viral vector	49
Bharat Biotech [Covaxin]	Inactivated	14
Sinopharm (Beijing) [Covilo]	Inactivated	93
Sinovac [CoronaVac]	Inactivated	56

*In March 2024, AstraZeneca began to phase out production and distribution of their COVID-19 vaccine as global demand for the product declined

In India, the government has approved 2 protein subunit vaccines, 2 mRNA vaccines, 6 non-replicating viral vector vaccines, a DNA vaccine, and an inactivated viral vaccine [18]. In China, the government has approved 4 inactivated viral vaccines, 2 subunit vaccines, and 2 non-replicating viral vector vaccines [19].

The COVID-19 vaccines continue to be closely monitored in the post-authorization phase to gather more data on their safety profiles [20]. In early 2021, regulatory authorities received reports of two serious adverse events—thrombocytopenia and thrombosis occurring in rare cases shortly after COVID-19 vaccination [21]. These cases were primarily associated with the adenoviral vector-based vaccines, such as the ChAdOx1 nCoV-19 (AstraZeneca) and Ad26.COV2.S (Janssen) vaccines. The condition, known as vaccine-induced thrombosis and thrombocytopenia (VITT), appears to be a rare side effect triggered by these vector-based COVID-19 vaccines.

The vaccines seem to induce autoantibodies that activate platelets, leading to the development of blood clots and low platelet counts [22]. In response to these safety concerns, several countries have temporarily paused or restricted the use of the ChAdOx1 and Ad26.COV2-S vaccines, especially in younger populations who may be at higher risk of developing VITT.

### Differences in vaccine response data between mRNA vaccines:

There are varying quality and quantity of evidence supporting the use of the mRNA vaccines in children. Given the fact that there is not a single vaccine that is being used across all jurisdictions, it is not practical for there to be a single preferred vaccine to be used in all jurisdictions. Among mRNA vaccines, post-marketing surveillance data have revealed differences in the antibody responses generated by the Moderna vaccine compared with the Pfizer BioNTech vaccine [23–26].

In addition, differences in side-effect profiles have been documented and have resulted in decisions within jurisdictions to use one product over the other in children [27]. For example, in Canada, the Pfizer BioNTech vaccine was stated as being preferred for use in children due to the observation of fewer cases of myocarditis compared with the Moderna vaccine [27]. On the other hand, the Moderna vaccine was regarded as being preferred among immunocompromised adults due to the likelihood of a more robust immune response [28].

1.2 **Complimentary vaccination strategy/strategies to use to mitigate the risk of severe outcomes from COVID-19 among children with kidney diseases**

### Recommendation:

We recommend using two complimentary strategies to reduce the risk of severe outcomes from COVID-19 among children with kidney diseases. First, children should be offered vaccination based on age-appropriate guidance within their respective jurisdiction. Second, persons caring for these children and family members should be vaccinated against COVID-19 (Grade B, Moderate Recommendation).

### Evidence and rationale:

In addition to direct vaccination of vulnerable persons, such as compromised children, it has been well-established that ring vaccination or the use of the cocoon strategy offers protection to these children [29]. This has been well-demonstrated for the prevention of several vaccine-preventable diseases as a means of protecting high-risk populations [30]. For these children, protection is largely based on the creation of a protective cocoon around them by vaccinating caregivers and household members.

For the COVID-19 vaccines, which are non-live products, there is no contraindication to vaccination with respect to the risk of vaccine-associated infection. However, for children who might not be able to mount adequate immune responses to the vaccines, the cocoon strategy offers protection and is highly recommended. In this regard, within jurisdictions, it is appropriate to prioritize vaccine access for persons who qualify as being part of a potential protective cocoon for vulnerable children.

1.3 **Primary COVID-19 vaccine series for children with kidney diseases**

### Recommendation:

We suggest that the exact number of recommended COVID-19 vaccine doses that define up-to-date vaccine status depends on the vaccine that is being used. Generally, a minimum of two doses are needed to complete a primary series. In general, for mRNA vaccines, a primary series might consist of 3 doses of vaccine among persons who are immunocompromised (Grade D, Weak Recommendation).

### Evidence and rationale:

Generally, two doses of COVID-19 vaccine have traditionally defined a primary series for most available vaccines. However, data from immunogenicity studies have indicated that some immunocompromised persons, including solid organ transplant recipients, require additional doses of the vaccine for optimal protection [31–33]. While data are lacking for children with varying degrees of the immunocompromised states associated with their underlying diseases, it is likely that those who are among the most immunocompromised are more likely to mount better immune responses after 3 doses compared to 2 doses [29, 30].

Given the paucity of pediatric data, it is necessary to examine relevant adult data and extrapolate to the pediatric population, where appropriate. A systematic review examined pooled evidence evaluating the humoral response of hemodialysis patients (HDP) post-mRNA SARS-CoV-2 vaccination [34]. The investigators performed a systematic search of the literature through MEDLINE, CINAHL, PubMed, EMBASE, and Web of Science databases, as well as medRxiv and bioRxiv preprint servers up to January 2023. Cohort and case–control studies were included if they compared the immune response between patients undergoing hemodialysis who received the mRNA SARS-CoV-2 vaccine and those who received the same vaccine but were not undergoing hemodialysis. From 120 studies identified, nine met the inclusion criteria (1969 participants). Hemodialysis patients developed lower antibody levels across all timepoints post-vaccination when compared with controls. Patients with CKD elicited the highest antibody immune response, followed by hemodialysis patients and then, kidney transplant recipients.

Overall, post-vaccination antibody titers were lower than in the healthy population. This is consistent with the findings of a systematic review and meta-analysis that showed that immunocompromised patients, notably transplant recipients, had lower immunogenicity with two doses of COVID-19 mRNA vaccines (assessed by seroconversion rates using anti-SARS-CoV-2 spike IgG after the second dose of COVID-19 mRNA vaccines). In this particular report, subgroup analysis based on type of transplantation (kidney vs. others) showed no statistically significant between-group differences in terms of immunogenicity [35]. In the absence of large-scale reports of SARS-CoV-2 vaccination in patients with nephrotic syndrome using immunosuppressive agents, investigators examined antibody responses in a small prospective study of 40 patients with nephrotic syndrome who were vaccinated at a median age of 16 years [36].

All patients mounted antibody responses to the vaccine with the median antibody titer being 598 U/mL (interquartile range, 89–1380 U/mL). Patients receiving mycophenolate mofetil (MMF) showed lower antibody titers than those who were not (median: 272 U/mL vs. 2660 U/mL, *p* = 0.0002). Serum immunoglobulin G (IgG) levels showed a weak linear relationship with antibody titers (R2 = 0.16). These data help to define patients who might be prioritized for additional doses of COVID-19 vaccines beyond the minimum number of doses that define series completion.

It should be noted that the use of the terminologies “primary series” and “booster” are being replaced as new updated vaccines emerge. For these vaccines, the number of doses depends on several factors, including age, vaccine type, and previous episodes of COVID-19 [29].

1.4 **The interval between the occurrence of SARS-CoV-2 infection and the initiation of COVID-19 vaccines**

### Recommendation:

We recommend that clinicians be guided by jurisdictional policies regarding the interval between SARS-CoV-2 infection and the administration of COVID-19 vaccines (Grade C, Moderate Recommendation).

### Evidence and rationale:

Guidance is based on expert opinion, considering immunological principles. The suggested interval between SARS-CoV-2 infection and the administration of COVID-19 vaccines depends on several factors, including but not limited to the extent to which the host is immunocompromised, the presence of previous multisystem inflammatory syndrome in children (MIS-C) and whether infection occurred before the completion or initiation of the primary vaccination series. Table 2 shows a suggested approach adapted from the interim guidance from the Canadian National Advisory Committee on Immunization, which does not represent an exclusive course of action [37].

**Table 2:** Intervals between SARS-CoV-2 infection and vaccination

Timing of SARS-CoV-2 infection relative to COVID-19 vaccination	Target population	Suggested intervals between SARS-CoV-2 infection and vaccination
Infection in individuals who have not been previously vaccinated or in those who are in process of completing a vaccination series	Individuals 6 months of age and older who are not considered moderately to severely immunocompromised and with no history of MIS-C or MIS-A^#^	Receive the vaccine 8 weeks after symptom onset or positive test (if asymptomatic)
	Individuals 6 months of age and older who are moderately to severely immunocompromised and with no history of MIS-C or MIS-A	Receive the vaccine dose 4 to 8 weeks after symptom onset or positive test (if asymptomatic)
	Individuals 6 months of age and older with a history of MIS-C or MIS-A (regardless of immunocompromised status)	Receive the vaccine dose when clinical recovery has been achieved or ≥ 90 days since diagnosis of MIS-C or MIS-A, whichever is longer
Infection in individuals who have been previously vaccinated	Individuals currently eligible for a fall 2023 COVID-19 dose(s)	Receive vaccine dose 3 – 6 months (84—168 days) after previous COVID-19 illness (characterized by positive test or after having symptoms post contact with someone who had a positive test)

^#^Multisystem inflammatory syndrome in adults

1.5 **Updating COVID-19 vaccines for children with kidney diseases**

### Recommendation:

We suggest that children receive updated COVID-19 vaccines as aligned with local jurisdictional policies and as guided by age-appropriate considerations (Grade C, Weak Recommendation).

### Evidence and rationale:

Doses given beyond the primary series were previously regarded as booster doses. These doses were deemed necessary due to waning immunity after the primary series, resulting in reinfections [38, 39].

In addition, attenuated responses occur among immunocompromised patients, including children with CKD. A standard COVID-19 mRNA vaccine regimen in immunosuppressed pediatric kidney transplant recipients (KTR) and CKD patients elicited an attenuated humoral immune response [40]. Only 62.3% of KTR and 80.8% of the CKD patients on immunosuppressive therapy sero-responded compared to 95% of the patients with CKD without immunosuppressive medication. The magnitude of the humoral immune response in KTR was ninefold lower than in the patients with CKD without immunosuppressive medication [40].

The timing of booster doses was dependent on several factors including the age of the child and the presence or absence of immunocompromised states. The use of boosters varied across jurisdictions. In countries where mRNA vaccines were used, bivalent boosters were preferred. Bivalent vaccines that target the BA.1 and BA.4/5 sublineages of Omicron were previously available [41] but have been replaced by a monovalent vaccine corresponding to Omicron variant XBB.1.5 of SRAS-Cov-2 [42, 43].

The precise makeup of future updated vaccines will depend on the prevalent circulating lineages. In addition, the number of updated doses required depends on age, vaccine type, whether or not a child previously had COVID-19, and the number of doses of vaccines they previously received [44].

1.6 **SARS-CoV-2 antibody testing for informing vaccination requirements for children with kidney diseases, including those who are immunocompromised**

### Recommendation:

There is insufficient evidence on the role of antibody testing in routine clinical practice as a means of informing vaccination strategies for children with kidney diseases.

### Evidence and rationale:

The full range of immune markers that define immunity against SARS-CoV-2 infection and disease remains to be determined. While many serologic tests that measure antibodies to the virus’ spike protein are being used around the world, the gold standard for evaluating immunity is the detection of neutralizing antibodies, as these have been shown to more accurately reflect the antibodies' ability to block the virus [45]. Studies have demonstrated a strong correlation between antibodies, both neutralizing and binding, and protection in clinical efficacy trials, with up to 90% of the variability in vaccine efficacy potentially explained by antibody levels [46, 47].

Therefore, it is reasonable to suggest that post-immunization antibody levels may be a useful measure of short-term protection. Data are emerging on which antibody levels may be associated with protection against different variants of SARS-CoV-2 and the World Health Organization (WHO) has proposed using Binding Antibody Units as a measure [48].

However, no specific antibody level has been identified as a correlate of protection against severe COVID-19 outcomes. Other immune markers, including mucosal immunity, cell-mediated adaptive immunity, and innate immunity, are thought to be important for immunity to SARS-CoV-2, though their individual or combined contributions to protection from the virus and its varying severity of disease are yet to be established [49, 50].

1.7 The role of hybrid immunity (natural SARS-CoV-2 infection plus vaccine) in protecting children with kidney diseases from severe COVID-19-related outcomes

### Recommendation:

While emerging data have shown that hybrid immunity is associated with enhanced antibody responses compared with responses that are solely induced by vaccines, there are insufficient data and pragmatic issues that prevent a recommendation of this approach as a formal public heath strategy to prevent severe outcomes among children with kidney diseases.

### Evidence and rationale:

COVID-19 hybrid immunity is an emerging concept that involves combining different types of immunological strategies to elicit protective immune responses to SARS-CoV-2. This in essence is the immune response profile provided by the combination of SARS-CoV-2 infection and COVID-19 vaccination [51]. Neutralizing antibody and memory B cell response elicited by a single dose of mRNA vaccine following previous infection with SARS-CoV-2 have resulted in an increased antibody titer that is approximately equivalent to a two-dose vaccine regimen in individuals who were not previously infected [52, 53].

In one study of healthcare workers vaccinated 7–11 months after infection with SARS-CoV-2, antibody titers measured 6 days following their first vaccination dose were twice as high as the antibody titers measured the month after their initial infection, and were able to neutralize wild-type, alpha, and beta variants, irrespective of vaccine type, number of doses, or pre-vaccination antibody titers [54].

Overall, the emerging data suggest that hybrid immunity might provide better protection against subsequent COVID-19 (symptomatic or severe) than either vaccination or infection alone, resulting in higher antibody levels, less frequent and less severe infection [51]. A systematic review and meta-regression indicated that hybrid immunity was associated with higher and more durable antibody responses compared with the non-hybrid state; however, the review identified limitations and the need for additional research [55].

1.8 **COVID-19 vaccination and its implication in the genesis of kidney disorders**

### Recommendation:

While case reports and registry data suggest a possible rare temporal association between COVID-19 vaccination and the onset of intrinsic kidney disorders, cause-and-effect relationships have not been definitively established, and the benefits of vaccination significantly outweigh potential risks among children with kidney disorders (Grade D, Weak Recommendation).

### Evidence and rationale:

There have been case reports and registry data of a temporal association between the administration of COVID-19 vaccines and the onset of some kidney disorders. A systematic review among children and adolescents examined reports in the English language literature and identified 18 cases of intrinsic kidney disorders from the published articles selected [56]. Among these, IgA nephropathy was most frequently reported, with 10 cases of IgA nephropathy (1 case of rapidly progressive glomerulonephritis requiring acute hemodialysis), 5 cases of minimal change disease (MCD), 1 case of concurrent MCD/tubulointerstitial nephritis (TIN) and 2 cases of TIN.

Data from the IRocGN2 International Registry suggests that there might be a temporal association between COVID-19 vaccination and IgA nephropathy (IgAN) and minimal change disease (MCD); however, causality was not established [57].

A review of IgA cases that were reported as being temporally associated with COVID-19 vaccination among adults and children identified 32 IgA nephropathy articles related to COVID-19 vaccines [58]. A total of 48 patients were diagnosed with IgA nephropathy by kidney biopsy, including 31 newly diagnosed IgA nephropathy (64.6%) and 17 relapsed IgA nephropathy (35.4%). Most patients were Asians (41.7%), followed by Americans (37.5%) [59]. It is possible that unreported temporal associations exist between vaccination and the onset of intrinsic kidney disorders in children. Although such temporal associations might exist, cause-and-effect relationships have not been definitively established.

In summary, the available data on the potential link between COVID-19 vaccination and the development of intrinsic kidney pathology in children and adolescents are currently limited. It is important to note that the number of reported cases of kidney pathology following COVID-19 vaccination is extremely small compared to the large number of vaccinations administered to this age group.

The benefits of COVID-19 vaccination far outweigh the potential risks. This is particularly important for children with pre-existing kidney disease, as they are at higher risk of complications from SARS-CoV-2 infection. Ongoing monitoring and reporting of cases, as well as further research, are necessary to gain a better understanding of any potential associations between COVID-19 vaccination and kidney pathology.

2. **Infection control measures for high/low-risk areas (e.g., HD units) and for patients with pre-existing kidney disease**
  Pediatric patients with CKD can be at a higher risk of contracting SARS-CoV-2 due to decreased immunocompetence and exposure to higher-risk environments. However, further, the activity of COVID-19 in a region can substantially influence the transmission and contraction of respiratory viruses. High SARS-CoV-2/COVID-19 activity areas are suggested to adhere to more specific guidelines.

2.1 **High SARS-CoV-2/COVID-19 Activity Areas: (Location poses a high risk of contracting the infection)**
2.1.1 **Office/clinic**
2.1.1.1 **In case of a hospital outbreak**

**Recommendations:**

- We suggest using telehealth video visits/phone calls (whatever is available) for children with kidney diseases and associated immunosuppressed state, to prevent transmission of SARS-CoV-2, and to monitor symptoms if SARS-CoV-2 positive (Grade C, Weak Recommendation).
- We suggest increasing the intervals between follow-up appointments in patients that are clinically stable (Grade D, Weak Recommendation).
- We recommend limiting patient companions to one caregiver for in-person visits (Grade X, Strong Recommendation).
- We suggest disinfecting the examining rooms after each patient (Grade C, Weak Recommendation).
- We suggest using electronic orders for prescriptions, if available (Grade C, Weak Recommendation).
- We suggest postponing non-urgent kidney biopsies until the local incidence and risk of infection decreases (Grade C, Weak Recommendation).
- We suggest developing a standard protocol of recommendations for parents/patients with potential exposure to COVID-19 in relation to local epidemiology (Grade C, Weak Recommendation).
- We recommend frequent monitoring of blood pressure at home for those on antihypertensive medication especially in patients having COVID-19 and having diarrhea (Grade C, Strong Recommendation).
- We suggest the utilization of urine dipsticks at home for monitoring proteinuria in nephrotic syndrome patients during episodes of fever, infections and/or suspected relapse (edema) (Grade C, Weak Recommendation).
- We recommend family members to wear surgical masks at home when infected with SARS-CoV-2 (Grade X, Strong Recommendation).

### Evidence and rationale:

COVID-19 has had a profound impact on the kidney community. Specifically, in the pediatric population, the onset of COVID-19 with pre-existing kidney diseases has been known to cause an increased risk of secondary outcomes ultimately causing a potential concern for increased risk of mortality and morbidity rates [60]. Amongst these outcomes, hypertension, hematuria, anemia, and elevated creatinine are the most common clinical manifestations associated with pediatric kidney disease patients [61].

Despite the presentation of secondary clinical outcomes, many pediatric kidney disease patients remain asymptomatic regarding primary COVID-19 symptoms such as fever and upper respiratory symptoms [62]. Morello et. al studied COVID-19 in children with idiopathic nephrotic syndrome (INS) and showed that in most of the 43 patients who developed COVID-19, symptoms were mild and recovery was very comparable to that of healthy pediatric patients [63].

Additionally, an ancillary cohort study consisting of 197 children and 63 young adults demonstrated that except for 3 individuals, all patients with INS had mild symptoms. Furthermore, kidney function remained stable without need for additional treatment. It was found that infection was associated with exposure at home (OR 15.4, 95% CI 6.9–34.2) and number of household members (OR 1.45, 95% CI 1.21–1.73) [3].

Finally, Melgosa et. al found that in 16 patients (6 with kidney failure, 3 KT, and 3 on chronic hemodialysis), the severity of symptoms was mild with fever and URS (upper respiratory symptoms) being the most common.

However, more notably, although basal glomerular filtration rate worsened in 3 patients, it recovered quickly with hydration and drug dose adjustment [64]. This furthers the evidence that COVID-19 in pediatric patients with kidney disease is relatively mild, yet kidney complications are possible and therefore adhering to clinical and preventive guidelines for limiting COVID-19 transmission is still vital.

The guidelines for office and clinic management are very similar to the guidelines for healthy pediatric patients but may be more relevant in patients with chronic illness and frequent hospital visits such as children with CKD and glomerular diseases. Guidelines for home monitoring of CKD patients and those on immunosuppressive drugs are more specific as these factors put CKD patients at higher risk of contracting COVID-19. General guidelines may apply to children who are not on immunosuppression, but more stringent guidelines are needed for children on immunosuppression. Hospital visits create a high-exposure environment as many hospitals have experienced clusters of infections in non-COVID-19 wards due to fewer transmission prevention methods [65].

Therefore, limiting unnecessary hospital visits for CKD patients when possible is recommended. To do this, home monitoring of blood pressure is advised in CKD patients, and prescriptions for home monitoring machines should be fulfilled.

Prescribing home blood pressure monitors for children with CKD is essential for early detection and management of hypertension, potentially slowing disease progression. However, in limited-resource settings, access to such equipment may be scarce. Consequently, patients often rely on local primary healthcare providers or other community health workers for regular monitoring [66, 67]. This highlights the need for community health initiatives to ensure all children with CKD have access to vital monitoring tools and medical support, regardless of their socio-economic circumstances.

Additionally, when necessary, dipstick urine tests that can be done at home to monitor proteinuria are suggested. For patients on immunosuppressive drugs, revised protocols for COVID-19 testing are advised as well as symptomatic tracking for upper respiratory symptoms and fever. Additionally, switching to oral drugs, when possible, can limit infection contraction from more invasive or intravenous injections for medication. It is essential to balance the need for infection prevention with the necessity of routine care, including immunizations, by implementing strategies such as scheduling dedicated clinic hours for high-risk patients, utilizing telemedicine when appropriate, and ensuring stringent infection control measures in healthcare settings to limit hospital visits during outbreaks.

2.1.2 **Hemodialysis unit**

**Recommendations**:

- We recommend sharing patient education about COVID-19 and precautionary measures in dialysis units with patients and their families stressing hygiene (Grade C, Moderate Recommendation).
- We recommend mandating respiratory viral screening including COVID-19 (Grade C, Moderate Recommendation).
- We recommend that all patients, accompanying family members and staff, wear surgical masks (Grade C, Moderate Recommendation).
- We recommend constant and consistent disinfection of dialysis machine post-treatment, which should be conducted using a 500 mg/L chlorine disinfectant for more than 30 min (this includes any surfaces, alternative equipment, setting where blood, secretions, contamination is present) in patients with COVID-19 (Grade C, Moderate Recommendation).
- We recommend using a fixed dialysis machine for COVID-19 patients (Grade C, Moderate Recommendation).

### Evidence and rationale:

Pediatric patients with COVID-19 who require hemodialysis (a high SARS-CoV-2/COVID-19 activity setting) pose a unique and high-risk obstacle. Additionally, as fewer hemodialysis units are present in less populated regions, the need for the patient to travel can increase the risk of COVID-19 as they travel through different regions of COVID-19 prevalence. Beyond logistical consequences, the facility set-up can increase risk as well.

Most hemodialysis units are filled with numerous patients, family members providing support, and staff in a confined setting, with limited isolation rooms [68].

A single-center study conducted in Wuhan, China saw 42 out of 230 HD patients (18.26%) and 4 out of 33 medical staff (12.12%) contract COVID-19, which was a higher contraction rate than the general population at the time [69].

A study by Canpolat et al. provides evidence that most pediatric patients on dialysis are more likely to contract COVID-19; however, they experience asymptomatic or mild symptoms and usually with a favorable outcome [70]. The study included 17 pediatric patients on dialysis with a positive PCR test for COVID-19, where 15/17 were asymptomatic or had a mild disease, 15/17 required no respiratory support, and 16/17 fully recovered.

However, we should also note that 14/17 required hospitalization in this single center study. Thus, although the course of COVID-19 is mild in pediatric dialysis patients with kidney disease, hospitalization rates can be high which can increase risk of exposure and additional secondary complications. Our clinical practice recommendations for pediatric patients in hemodialysis units mostly align with our COVID-19 prevention recommendations above for pediatric CKD patients with additional preventative facility guidelines as the risk of infection is higher in these patients. As hemodialysis units create high-exposure environments for pediatric patients, limiting the spread of COVID-19 within these clinics is of utmost importance. Preventative measures should be taken extremely seriously such as wearing PPE and limiting extra personnel in the area, as these have been common causes of higher transmission rates [71].

Additionally, utilizing efficient hemodialysis schedules to disperse and distance patients as much as possible can limit spread as well [72]. As patients enter the clinic, safety measures can be taken such as symptomatic tracking and fever monitoring to minimize the risk of COVID-19 transmission prior to their dialysis appointment [73]. The machines within a hemodialysis unit also add to the risk of transmission due to exposure to bloodborne pathogens and transmissible secretions. Thus, we suggest that all machines and equipment be wiped down using 500 mg/L chlorine disinfectants to ensure cleanliness among patients [74].

Moreover, assigning each patient to a machine and keeping this constant between every appointment will mitigate the spread between machines and ensure limited transmission if a patient were to be infected [74]. Lastly, educating both patients and parents on the risks and preventative protocols in place to limit the spread of COVID-19 can help increase awareness and safe practices at home as well for pediatric patients at higher risk.

Overall, it is evident through critical analysis that the clinical course of COVID-19 in pediatric patients with pre-existing kidney disease in home, hospital, and hemodialysis units is mild and comparable to the healthy pediatric population. However, adverse complications and severe outcomes can still be observed on a case-by-case basis. Thus, ample preventive measures, especially in the HD unit (where transmission is high), should be taken by clinicians and patients to improve care, recovery, and quality of life for all members.

2.2 Areas with low SARS-CoV-2/COVID-19 activity

Pediatric patients in areas/settings with low SARS-CoV-2/COVID-19 activity are encouraged to follow less stringent guidelines. This is true of patients with early-stage CKD and no need for hemodialysis, which can limit the frequency of clinical exposures and hospital visits where COVID-19 transmission is high [75]. These regions may abide by more standard masking protocols for COVID-19 protection.

Furthermore, for patients on peritoneal dialysis (PD), the risk of COVID-19 transmission can be reduced as PD patients are trained to perform dialysis operations at home, isolated from dialysis centers and in-hospital visits.

However, due to the potential abnormal immune function of children on PD, PD-specific guidelines must be followed by caregivers and patients at home to ensure safe dialysis treatment and to prevent increasing the risk of COVID-19. Training caregivers within the patient’s family and home is ideal to limit the amount of external and clinical exposures needed if PD is conducted by caregivers outside of the patient's circle. That said, if caregivers are positive for COVID-19, it is imperative that they do not expose the patient and wear appropriate PPE including gloves, surgical mask, and protective accessories when near the patient.

Additionally, PD fluid should be double-bagged and appropriately labeled prior to sending it to the lab to limit any potentially infectious spills. Patients needing to consult with medical professionals should first be assessed via telehealth to limit the amount of in-person clinical exposures.

Furthermore, they will be virtually screened for any symptoms of COVID-19 or other acute respiratory illnesses [76]. These recommendations provide correct protective measures in low SARS-CoV-2/COVID-19 activity settings which should be taken especially if the patient does need to be seen in a hospital or clinic to ensure a safe treatment regimen.

2.2.1 Standard precautions

**Recommendations**:

- We recommend limiting exposure to caregivers or individuals who have suspected or confirmed COVID-19 or any other type of respiratory illness (Grade C, Moderate Recommendation).
- We recommend frequent disinfecting of patients’ living areas, especially shared areas, and high-touch surfaces (Grade C, Moderate Recommendation).
- We recommend family members to wear surgical masks at home when infected with SARS-CoV-2 (Grade C, Moderate Recommendation).

2.2.2 PD specific

**Recommendations:**

- We recommend conducting PD in an area or room exclusive for this procedure (Grade C, Moderate Recommendation).
- We recommend wearing PPE by all caregivers if the patient is positive for COVID-19 or cannot do the procedure by themselves (Grade C, Moderate Recommendation).
- We suggest conducting PD management appointments via telehealth whenever possible, to limit clinical exposure (Grade C, Weak Recommendation).
- We suggest collecting PD fluid at home for testing whenever possible and prescriptions for this should be sent via electronic attachment (Grade C, Weak Recommendation).
- We suggest that PD fluid always be treated as an infectious biohazard and disposed of accordingly, similar to protocol for patients positive for HIV (Grade C, Weak Recommendation).

2.3 COVID-19 episodes

Regardless of high/low SARS-CoV-2/COVID-19 activity regions, COVID-19 can be contracted, in which specific and prompt action by the caregiver is imperative to prevent further transmission. We recommend immediate isolation and PPE steps to mitigate the spread of the virus, especially in dialysis units. Specific details on recommendations are provided below [77].

2.3.1 For patients on hemodialysis

**Recommendations**:

- We suggest educating staff on proper PPE donning and doffing during the treatment of COVID-19-positive patients (Grade C, Weak Recommendation).
- We recommend suspected or positive COVID-19 patients wear a disposable three-layer surgical mask throughout the entire dialysis duration (Grade C, Moderate Recommendation).
- We recommend dialyzing patients with positive COVID-19 or symptoms in a room exclusively for positive patients with a closed door. If this is not possible, consolidating all positive patients to dialyzing during the same shift is beneficial to avoid further transmission (Grade C, Moderate Recommendation).

2.3.2 For non-dialysis patients

### Recommendation:

Patients with suspected or positive COVID-19 should be followed by a COVID-19 care team and follow standard isolation protocols recommended by the local policies and as guided by age-appropriate considerations (Grade C, Moderate Recommendation).

3. Precautions for patients undergoing transplantation

**Recommendations**:

- We recommend that children on the kidney transplant waiting list receive the COVID-19 vaccination if they are eligible according to their local public health authority's guidelines, as part of their pre-transplant vaccination program (Grade C, Moderate Recommendation).
- We recommend that living kidney donor candidates be vaccinated against COVID-19 if they are eligible based on the recommendations of their local public health authority (Grade C, Moderate Recommendation).
- We suggest testing the SARS-CoV-2 serology of the recipient to assess the response to vaccination. We suggest that additional vaccine doses be administered if there is no response after the initial vaccination (Grade C, Weak Recommendation).
- We suggest that the recipient and the living donor candidate self-isolate/refrain from gathering for 14 days before the planned transplant surgery to minimize the risk of exposure, especially in high-risk areas or in non-vaccinated subjects (Grade C, Weak Recommendation).
- We recommend that the recipient, living donor, and caregivers use protective equipment such as surgical masks, to reduce the risk of exposure to the virus (Grade C, Moderate Recommendation).
- We recommend screening the recipient and the donor for COVID-19 symptoms and by nasal swab PCR before the transplant surgery (Grade C, Moderate Recommendation).
- We recommend delaying LDTx in case of ongoing COVID-19 in the donor or recipient (Grade C, Moderate Recommendation).
- We suggest, for deceased donors with COVID-19, that the transplant center determine the suitability of kidney donation based on individual patient needs and local allocation policy (Grade C, Weak Recommendation).

### Evidence and rationale:

Children appear to be less frequently and less severely affected by COVID-19 than adults, both in the general population and in immunocompromised patients [78–80]. In published series, very few children with kidney transplants have been admitted to intensive care or died from COVID-19 [64, 79, 80].

An international cohort of 113 children immunocompromised due to kidney disease, including 53 kidney transplant recipients, infected with SARS-CoV-2 between March and July 2020 reported less severity than that observed in adult counterparts with only 5% requiring bi-level positive airway pressure or ventilation [78].

However, 4/113 children died in low-income countries. In a subsequent large North American study, 465 pediatric kidney transplanted children were tested for COVID-19 between September 2020 and February 2021 and 109 (23%) were positive [64]. Most patients required only symptomatic outpatient treatment, 16% were hospitalized and 5% were in intensive care. One case of respiratory failure and one death were attributed to COVID-19. In a study of 200 transplant recipients from Italy, none of the patients developed COVID-19 pneumonia.

Additionally, there was no increased risk of severe disease or mortality in these patients. Based on these findings, the author hypothesized that instead of amplifying the risk of COVID-19 in recipients, immunosuppression may actually be protective [81]. The rationale is that immunosuppression may dampen the amplified immune response that can contribute to severe COVID-19 disease progression.

Other post-transplant complications have been reported in rare cases, such as AKI, allograft rejection [79], de novo collapsing focal segmental glomerulosclerosis [82] or post-viral allograft vasculitis in cases of recent COVID-19 in the donor or recipient [83].

Conversely, one case has been reported of uneventful kidney transplantation in an asymptomatic child with a positive nasal swab PCR but low viral load on the day of surgery treated with standard immunosuppression protocol [84].

Vaccination appears to be safe in children. In the literature, the immunogenicity of SARS-CoV-2 vaccines is lower in transplanted children than in healthy children, and moderately higher than in transplanted adults [33, 85]. Risk factors for nonresponse include recent transplantation < 3 years, antimetabolites, or multiple immunosuppressive drugs, delta and the omicron variants compared to the wild-type strain.

Therefore, we recommend vaccinating children before transplantation. Additional vaccine doses may be necessary for children who do not respond after two doses. We advocate the development of vaccines specific to the strains currently in circulation.

The safety of kidney transplantation from donors with recent or current SARS-CoV-2 infection is an area of active research. Recent studies have suggested that in carefully selected cases, such transplants can be performed safely with appropriate precautions. However, there are still many open questions, and the guidance continues to evolve as more data become available. Ultimately, the decision to proceed with such a transplant requires a careful assessment of the risks and benefits for each individual case in consultation with the transplant team [86–89].

4. Managing children with suspected or confirmed COVID-19 while on immunosuppression
5. Supportive care

**Recommendations**:

- We recommend an assessment of the severity of the COVID-19 presentation (Grade C, Moderate Recommendation).
- We recommend supportive care in most instances of presentations for children with kidney disease on immunosuppression (Grade C, Moderate Recommendation).
- We recommend careful fluid monitoring in children who are admitted, and appropriate fluid administration to treat dehydration and associated acute kidney injury (Grade C, Moderate Recommendation).

### Evidence and rationale:

While COVID-19 has shown the propensity for rapid spread, pediatric patients with conditions such as CKD are especially vulnerable [90]. Glomerulonephritis on immuno­suppression [91, 92], chronic dialysis [93], atypical hemolytic uremic syndrome [94], and those living with a kidney transplant [95–97], can be both at much higher risk for being infected and suffer from worse clinical outcomes as compared to healthy peers.

Based on recent national registry data from Mexico, the overall mortality rate in patients < 18 years of age was 1.3% with risk factors including obesity, hypertension, diabetes, chronic lung disease, and kidney disease [98]. Another Mexican pediatric cohort with 131,001 patients showed an overall mortality rate of 0.6% and a rate of 7.28% for the subset with CKD (OR = 13.250, 95% CI, 9.066–19.350) [99].

Similar findings were observed among US adolescents [100]. One small study from the US suggested that immunocompromised kidney transplant patients may have similar outcomes compared with immunocompetent children [101] and was found in a population of 5 transplant centers in the US [96]. Further evaluation of 55,924 laboratory-confirmed COVID-19 cases in China showed that factors such as the presence of dyspnea, respiratory rate ≥ 30/min, blood saturation levels ≤ 93%, PaO2/FiO2 ratio ≤ 300, lung infiltrates ≥ 50% of the lung fields between 12–48 h were associated with severe COVID-19 [102].

In a US pediatric registry of COVID-19 cases, approximately 25% of pediatric kidney transplant patients were asymptomatic. The most common symptoms seen were cough, fever, dry/sore throat, and rhinorrhea. A small proportion of these patients also presented with chest pain, anosmia, diarrhea, or headache. While approximately 30% of patients required hospitalization, the majority did not require supplemental oxygen, intubation, or extracorporeal membrane oxygenation [96]. While the anxiety levels of these patients were not different from their healthy peers [103], their caregivers experienced significantly more anxiety and depression [104]; suggesting the need for special support and counseling.

The most common treatment modality was often supportive care, even among infants on immunosuppression [105]. Provision of supportive care using acetaminophen is recommended for all ages.

4.2 Specific treatment for COVID-19 Illness

**Recommendations**:

- We recommend immunosuppressed children who have an increasing oxygen requirement be given dexamethasone or similar/equivalent steroid (Grade B, Strong Recommendation).
- We suggest immunosuppressed children who have a high risk of worsening disease but are not requiring oxygen be given remdisivir within 7 days of symptom onset (Grade C, Weak Recommendation).
- We recommend prophylactic anticoagulation in adolescents hospitalized with COVID-19 (Grade D, Weak Recommendation).
- We recommend administering specific antiviral treatment to patients showing signs and symptoms indicative of respiratory failure, septic shock, or multiple organ dysfunction/failure (Grade C, Moderate Recommendation).
- We suggest immunosuppressed children who have an oxygen requirement be given remdesivir (Grade C, Weak Recommendation).
- We suggest immunosuppressed children requiring mechanical ventilation or extracorporeal membrane oxygenation with no improvement on dexamethasone be given baricitinib or toxilizumab (Grade C, Weak Recommendation).
- We suggest that immunosuppressed adolescents (aged over 12 years and weighing more than 40 kg) at high risk of severe infection be treated with nirmatrelvir-ritonavir within 5 days of symptom onset (Grade C, Weak Recommendation).
- We recommend dosing adjustment of nirmatrelvir-ritonavir in CKD (Grade C, Moderate Recommendation).
- We recommend close monitoring and cautious use of nirmatrelvir-ritonavir with cyclosporine, tacrolimus, and everolimus due to drug interactions (Grade C, Moderate Recommendation).
- We recommend dose adjustment of nirmatrelvir-ritonavir in combination with atorvastatin and rosuvastatin (Grade B, Moderate Recommendation).
- We recommend nirmatrelvir-ritonavir not be used with amiodarone, colchicine, and simvastatin (Grade C, Moderate Recommendation).

### Evidence and rationale:

Clinical signs indicative of respiratory failure, septic shock, or multiple organ dysfunction/failure were associated with worse disease course and poor prognosis [102]. The effect of commonly used drugs on mortality and the average length of hospital stay of COVID-19 patients are as follows:

- **Dexamethasone**
  A meta-analysis of two major RCTs with more than 6,000 adult patients with moderate to severe COVID-19 showed 10% reduction in mortality with dexamethasone compared to standard care [106].
  A study in adult patients, and not specific to individuals with CKD. The 7-day fixed-dose hydrocortisone course was terminated early, resulting in an underpowered study, but it demonstrated a 93% probability of benefit for fixed-duration dosing and an 80% probability of benefit for shock-dependent dosing, compared to no hydrocortisone, in improving the odds of organ support-free days within 21 days [107].
- **Remdesivir*(*intravenous infusion antiviral, Veklury®)**
  Remdesivir was associated with a considerably lower risk of mortality compared to tocilizumab, and it also resulted in a shorter time to clinical improvement compared to both hydroxychloroquine and tocilizumab [106].
  Five randomized controlled trials (3/5 open label of moderate to high risk of bias) evaluating remdesivir compared to placebo or comparing varying doses of remdesivir showed daily treatment with 100 mg remdesivir was no better than no treatment/placebo [108].
  A meta-analysis of 7 RCTs (4 overlapping with the above) showed improved clinical recovery rate on days 7, 14 and 28. Still, there was no significant mortality difference between the remdesivir and placebo groups at day 28. Notably, the remdesivir group had a lower risk of mortality than placebo at day 14, suggestive of a better effect with earlier initiation of remdesivir [109].
  Five days of remdesivir had significantly better side effect profiles than 10 days [110].
  Remdesivir can be given to children 28 days and older and weight at least 3 kg. The drug must be started within 7 days of symptoms [111].
  There is insufficient evidence to recommend either for or against the routine use of remdesivir. Consider treatment based on age and other risk factors.
- **Nirmatrelvir-ritonavir (oral antiviral, Paxlovid.®)**
  Nirmatrelvir-ritonavir is a safe and efficacious agent for COVID-19 [112, 113].
  A meta-analysis of 12 studies (2 RCTs, 10 observational studies) showed nirmatrelvir-ritonavir reduced mortality (RR 0.24; 95% CI, 0.15–0.37, I2 = 48%, moderate certainty), and hospitalization (RR 0.41; 95% CI, 0.29–0.59, I2 = 90%, low certainty) [112]. Subgroup analysis showed that the reduction in risk of hospitalization was slightly more pronounced in the patients without previous immunity to SARS-COV-2 than in patients already having immunity to SARS-COV-2.
  A larger meta-analysis of 23 studies (7 overlap with the above) showed nirmatrelvir-ritonavir improved mortality risk compared to no-nirmatrelvir-ritonavir (OR = 0.25; 95% CI, 0.14–0.45; *p* < 0.05) [113]. Hospitalization rate was reduced in the nirmatrelvir-ritonavir group (OR 0.4; 95% CI, 0.24–0.69; *p* < 0.05). Subgroup analysis on variables such as COVID-19 vaccination status was not able to be performed due to insufficient data.
  Meta-analysis of 3 studies did not find significant difference of COVID-19 rebound in patients treated with nirmatrelvir-ritonavir versus not (OR = 0.84; 95% CI, 0.67–1.04, *p* = 0.11) [113].
  It is licensed for 12 years and older, with a weight > 40 kg. It must be started within 5 days of symptoms. This approach has the highest level of evidence (B3) in adolescents.
- **Baricitinib**
  Baricitinib, an IL-6 receptor antibody, is a Janus kinase 1/2 inhibitor with known anti-inflammatory and anti-viral properties. A meta-analysis of 4 studies with 10,815 patients, showed statistically significant reduction in 28-day mortality in hospitalized patients who received baricitinib vs. standard care or placebo (OR 0.69, 95% CI, 0.50–0.94; *p* = 0.04, I2 = 65%) [114].
- **Tocilizumab**
  A meta-analysis of 6 RCTs showed no significant reduction in mortality by day 28–30 with the use of tocilizumab among hospitalized patients relative to those not treated with tocilizumab [115].
- **Neutralizing monoclonal antibodies (bamlanivimab/etesevimab, casirivimab/imdevimab, regdanvimab, sotrovimab)**
  Neutralizing monoclonal antibodies share a common mechanism of action, which is binding to and neutralizing the SARS-COV-2 virus.
  Four studies were included in a Bayesian network meta-analysis, 2 of which excluded patients who had received COVID vaccination. Less than half the publications of the above trials were peer-reviewed.
  Primary analysis (excluding regdanvimab) showed a statistically significant reduction in risk of hospitalization associated with all nMAB therapies versus placebo in patients who were exposed to SARS-CoV-2, who were at high risk of developing severe COVID-19 disease [116]. The meta-analysis estimated that treating 1000 patients with nMABs would prevent approximately 37 hospitalizations, 10 ICU admissions, 4 invasive ventilation events and 5 deaths, compared with placebo. The estimates were subject to considerable uncertainty.
- **Hydroxychloroquine (not recommended)**
  A Cochrane Systematic Review found 12 RCTs involving 8560 participants from China, Brazil, Egypt, Iran, Spain, Taiwan, UK, and North America, and a global study. Hydroxychloroquine had no effect on the risk of death from COVID-19 [117]. Hydroxychloroquine also has little/no effect on progression to mechanical ventilation and increases the risk of adverse events three-fold [117].
- **Oseltamivir**
  Eight observational studies were included in a meta-analysis which found inconclusive outcomes for oseltamivir versus a varying range of controls (lopinavir/ritonavir, hydroxychloroquine, ribavirin, IVIG, infliximab, chloroquine, azithromycin, prednisone/unnamed steroids, naproxime, Arbidol, non-use) [118].
- **Molnupiravir (oral antiviral, Lagevrio®)**
  Molnupiravir is only licensed for adults. The drug must be started within 5 days of symptoms.
- **Prophylactic anticoagulation:** is recommended for hospitalized adolescents [119].

Despite no comparative clinical trials evaluating prophylactic anticoagulation for COVID-19 in children having been published, there are multiple observational studies and meta-analyses that have been published. Moreover, given the lack of difference between healthy peers and kidney patients < 18, general treatment guidelines should apply [120–122]. The NIH recommended treatment for children with COVID-19 [119].

4.3 Dose adjustment for CKD and drug interactions
4.3.1 Nirmatrelvir-ritonavir

If eGFR > 60 mL/min/1.73 m^2^, nirmatrelvir-ritonavir should be used twice daily. For adults and adolescents, for each session, three tablets must be taken (two tablets with 150 mg of nirmatrelvir each and one tablet with 100 mg ritonavir [123]. If eGFR is 30—< 60 mL/min/1.73 m^2^, dose adjustment is required. There are still two doses per day, but only one 150 mg nirmatrelvir tablet is taken. Since these directions are subject to errors, it is recommended that written directions are provided and – if possible – the tablet that is to be omitted is marked. Since nirmatrelvir is excreted through the kidney, it should not be used if eGFR is < 30 mL/min/1.73 m^2^ [30]. However, the product monograph does not list this as a contraindication and there is insufficient evidence in the literature. A Canadian group suggests the following dosing based on a small study of 15 patients (Table 3) [124].

**Table 3:** Dosing of nirmatrelvir/ritonavir in children with eGFR < 30 mL/min and on hemodialysis

	Day 1	Day 2	Day 3	Day 4	Day 5
eGFR < 30 mL/min	1 × 300 mg Nirmatrelvir (= 2 tablets) + 1 × 100 mg Ritonavir (= 1 tablet)	1 × 150 mg Nirmatrelvir (= tablets) + 1 × 100 mg Ritonavir (= 1 tablet)	Like day 2	Like day 2	Like day 2
Hemodialysis	On dialysis days AFTER dialysis				
≥ 40 kg	1 × 300 mg Nirmatrelvir (= 2 tablets) + 1 × 100 mg Ritonavir (= 1 tablet)	1 × 150 mg Nirmatrelvir (= tablets) + 1 × 100 mg Ritonavir (= 1 tablet)	Like day 2	Like day 2	Like day 2
< 40 kg	1 × 150 mg Nirmatrelvir (= tablets) + 1 × 100 mg Ritonavir (= 1 tablet)	No application	1 × 150 mg Nirmatrelvir (= tablets) + 1 × 100 mg Ritonavir (= 1 tablet)	No application	1 × 150 mg Nirmatrelvir (= tablets) + 1 × 100 mg Ritonavir (= 1 tablet)

4.3.2 **Nirmatrelvir/ritonavir “use in children requiring immunosuppression”**

There are multiple drug-drug interactions with other medications that must be considered [125–132]. Extreme caution should be used when considering the use of nirmatrelvir/ritonavir in transplant patients.

The product monograph suggests careful monitoring of cyclosporine, tacrolimus and everolimus levels [123]. Since ritonavir is a strong CYP3A4 inhibitor as well as an inhibitor of P-glycoprotein, one must expect significant rise of cyclosporine, tacrolimus and everolimus levels within days of administration.

Several case reports mention substantial increases of tacrolimus levels associated with allograft dysfunction [133–137]. Several strategies were suggested to address the issue, including rifampicin [136] or complete halting of tacrolimus dosing [138]. We found no literature on strategies to overcome this problem in pediatrics. Essentially, close monitoring (even daily) of calcineurin or mTOR inhibitors is recommended. There are many open questions such as unknown bioavailability of calcineurin inhibitors at typical concentrations in combination with nirmatrelvir/ritonavir [139], or the timeline to normalization of the levels after discontinuation of nirmatrelvir/ritonavir. Based on this, extreme caution is to be used when considering nirmatrelvir/ritonavir in transplant patients on calcineurin or mTOR inhibitors, and their use is not recommended.

There are other drug-drug interactions with nirmatrelvir/ritonavir, and it is contraindicated with amiodarone, colchicine, and simvastatin, while dose adjustments are needed in combination with atorvastatin and rosuvastatin [123, 140]. Nirmatrelvir cannot be used alone since this drug alone is not effective against COVID-19.

4.3.3 **Molnupiravir**

There are no known drug-drug interactions with molnupiravir [124], but it is not licensed for < 18 years of age. This drug is considered less effective than nirmatrelvir/ritonavir but may be considered as an alternative.

5. **Special consideration for less-resourced countries (LRC) (applicable to all previous recommendations)**

In the available literature, the difficulties faced by less-resourced countries (LRC) as regards the management of pediatric patients with pre-existing kidney diseases during COVID-19 are very rarely addressed.

It seems reasonable to suggest that control measures, vaccination policies, and precautions needed in the setting of kidney transplantation should not be different than the ones recommended for high-resourced countries (HRC), even if it is more than obvious that the major issue in providing recommended care is the availability of the vaccines and the supply of protective equipment, and the necessary materials as stipulated in this guideline. It is still debated whether the prognosis of the infection and its influence on the management of the child, especially the immunosuppressive treatment, is worse for children living in LRC vs. HRC.

There are few series published describing the clinical course and the outcome of children with SARS-CoV-2 infection living in LRC. One of the more comprehensive studies comes from Brazil [141] and describes the medical outcomes and the risk factors for COVID-19-related fatality in a large cohort of hospitalized children with kidney diseases. The paper describes an analysis of a nationwide surveillance database of 21,591 hospitalized pediatric patients with COVID-19, between February 2020 and May 2021. Among these patients, 290 had kidney diseases and there was a mortality rate of 20.8% (59/290), compared to 7.5% (1602/21,301) in the non- kidney diseases cohort (Hazard ratio [HR] = 2.85, 95% CI, 2.21–3.68, *p* < 0.0001). It is important to underline that 51, 32, and 17% of the kidney diseased children had 1, 2, or 3 comorbidities, respectively, while the non-kidney diseased children had 19, 3, and 0.5%.

Another study, from India [61], retrospectively reviewed 88 children with CKD or nephrotic syndrome, in New Delhi from April 2020 to June 2021. The conclusion of the study was that these patients are at risk of AKI and of severe complications (mortality rate of 4.5%), particularly those children with nephrotic syndrome with SARS-CoV-2 infection during a relapse.

Considering these limited data, it appears that special attention has to be paid to those children living in socioeconomically disadvantaged regions with kidney diseases with one or more associated comorbidities and to the children who develop COVID-19 during a relapse of NS. In cases where dialysis is needed for MIS-C in LIC, peritoneal dialysis has been used successfully to treat AKI [142].

## Limitations of the guideline process

Managing children with pre-existing kidney disease during SARS-CoV-2 infection was a relatively new area of study at the time of the reported studies. As a result, some RCTs were of small numbers and of poor methodological quality, leading to most recommendations being classified as weak to moderate.

Additionally, no patient representatives were included in the guideline development group, which may have limited the consideration of patient perspectives and priorities in the guideline recommendations.

Future research should prioritize monitoring and addressing mental health concerns in pediatric patients with pre-existing kidney disease during seasonal outbreaks of COVID-19. This area was beyond the scope of the current clinical practice guidelines, yet it holds critical importance. The compounded stress from managing CKD alongside the heightened anxiety of a pandemic could significantly impact these children's psychological well-being. Investigating this intersection could reveal essential insights into holistic care strategies, ensuring that mental health support is integrated with medical treatment to improve overall health outcomes in this vulnerable population. Furthermore, we have identified future research recommendations to guide the next edition of this guideline (Supplementary Table S5).

## Supplementary Information

Below is the link to the electronic supplementary material.

Supplementary file1 (DOCX 30 KB)

Supplementary file2 (DOCX 83 KB)

## Abbreviations

COVID-19: Coronavirus disease of 2019
SARS-CoV-2: Severe acute respiratory syndrome coronavirus 2
MIS-C: Multisystem inflammatory syndrome
LIC: Low-income countries
IPNA: International Pediatric Nephrology Association
CKD: Chronic kidney disease
NS: Nephrotic syndrome
KRT: Kidney replacement therapy
AKI: Acute kidney injury
LRC: Less-resourced countries
RCTs: Randomized controlled trials
EUL: Emergency use listing
HDP: Hemodialysis patients
MMF: Mycophenolate mofetil
WHO: World Health Organization
PD: Peritoneal dialysis
PPE: Personal protective equipment
HIV: Human immunodeficiency virus
CDC: Centers for Disease Control and Prevention
PCR: Polymerase chain reaction
