# Supplementary material for: IPNA clinical practice recommendations on care of pediatric patients with pre-existing kidney disease during seasonal outbreak of COVID-19
Source: Pediatr Nephrol. 2024 Dec 29;40(5):1795–815. doi: 10.1007/s00467-024-06565-5 (PMC11946955; doi:10.1007/s00467-024-06565-5)
Supplement: Supplementary file 1 — Supplementary file1 (DOCX 30 KB) [file 467_2024_6565_MOESM1_ESM.docx]

**IPNA Clinical Practice Recommendations on Care of Pediatric Patients with Pre-Existing Kidney Disease During Seasonal COVID-19**

**on behalf of the International Pediatric Nephrology Association (IPNA)**

**SUPPLEMENTARY MATERIAL:**

**CONTENT:**

**Table S1 Area or expertise and responsibilities of core group members.**

**Table S2 Evidence Table of included studies with assigned aggregate evidence quality**

**Table S3 Keywords and MeSH terms for COVID-19 in children with KD used for literature search in PubMed database.**

**Table S4 Key health questions (PICOs)**

**Table S5 Future research recommendations**

# **Table S1 Area or expertise and responsibilities of core group members.**

| **Name** | **Area of expertise** | **Responsibilities** |
| --- | --- | --- |
| **Khalid Alhasan** | Pediatric nephrology | Leading coordinator of this project. Coordination of work groups and the process of generating the manuscript. Drafting of recommendations and evidence text and grading of recommendations. Incorporation of suggestions from the core group members, external experts and voting group members into the manuscript and reviewing the manuscript before submission |
| **Rupesh Raina** | Pediatric and adult nephrology | Drafting of recommendations and evidence text and grading of recommendations and reviewing the manuscript before submission |
| **Olivia Boyer** | Pediatric nephrology | Drafting of recommendations and evidence text and grading of recommendations and reviewing the manuscript before submission |
| **Jean Koh** | Pediatric nephrology | Drafting of recommendations and evidence text and grading of recommendations and reviewing the manuscript before submission |
| **Melvin Bonilla-Felix** | Pediatric nephrology | Drafting of recommendations and evidence text and grading of recommendations and reviewing the manuscript before submission |
| **Sidharth Sethi** | Pediatric nephrology | Drafting of recommendations and evidence text and grading of recommendations and reviewing the manuscript before submission |
| **Yasser Amer** | General pediatrics  Guideline methodology | Co-coordinator of this project. Literature search and creation of evidence tables, drafting of  recommendations and evidence text and grading of recommendations. Drafting of recommendations and evidence text and grading of recommendations and reviewing the manuscript before submission |
| **Paula Coccia** | Pediatric nephrology | Drafting of recommendations and evidence text and grading of recommendations and reviewing the manuscript before submission |
| **Mohamad-Hani Temsah** | Pediatric critical care, Research methodology | Drafting of recommendations and evidence text and grading of recommendations and reviewing the manuscript before submission |
| **Judith Exantus** | Pediatric nephrology | Drafting of recommendations and evidence text and grading of recommendations and reviewing the manuscript before submission |
| **Samina Khan** | Research Methodology | Co-coordinator of this project.  Organization of Delphi process.  Contributing to the writing up of the manuscript and tables. |
| **Xuhui Zhong** | Pediatric nephrology | Drafting of recommendations and evidence text and grading of recommendations and reviewing the manuscript before submission |
| **Vera Koch** | Pediatric nephrology | Drafting of recommendations and evidence text and grading of recommendations and reviewing the manuscript before submission |
| **Ali Duzova** | Pediatric nephrology | Drafting of recommendations and evidence text and grading of recommendations and reviewing the manuscript before submission |
| **Anil Vasudevan** | Pediatric nephrology | Drafting of recommendations and evidence text and grading of recommendations and reviewing the manuscript before submission |
| **Mignon McCulloch** | Pediatric nephrology | Drafting of recommendations and evidence text and grading of recommendations and reviewing the manuscript before submission |
| **Upton Allen** | Pediatric infectious diseases | Drafting of recommendations and evidence text and grading of recommendations and reviewing the manuscript before submission |
| **Guido Filler** | Pediatric nephrology and pharmacology | Drafting of recommendations and evidence text and grading of recommendations and reviewing the manuscript before submission |
| **Giovanni Montini** | Pediatric nephrology | Co-coordinator of this project.  Drafting of recommendations and evidence text and grading of recommendations and reviewing the manuscript before submission |

# **Table S2 Evidence Table of included studies with assigned aggregate evidence quality** *(in a separate Supplementary file)*

# **Table S3 Keywords and MeSH terms for COVID-19 in children with KD used for literature search in PubMed database.**

| **Keywords** | Pediatric nephrology, COVID-19, Pediatric patients, Kidney disease, Clinical practice recommendations, Seasonal variations, Pediatric care, Viral infections, Pediatric nephrologists, Disease management, SARS-CoV-2, Renal function, Pediatric infectious diseases, Comorbidity, Renal complications, Immunocompromised children, Pediatric critical care, Acute kidney injury, Seasonal illnesses, Pediatric renal care |
| --- | --- |
| **MeSH (Medical Subject Headings) Terms** | Pediatrics, Nephrology, COVID-19, Child, Kidney Diseases, Practice Guidelines as Topic, Patient Care, Virus Diseases, Pediatricians, Disease Management, SARS-CoV-2, Renal Insufficiency, Infectious Disease Medicine, Comorbidity, Critical Care, Acute Kidney Injury, Immunosuppression, Renal Replacement Therapy, Seasonal Variation |

# **Table S4 Key health questions (PICOs)**

| **Key questions** |
| --- |
| PICO 1: What is the best practice for COVID-19 vaccination in children with kidney disease and immunocompromised (e.g., CKD, NS) or with co-morbidities? This includes vaccinated family members. |
| PICO 2: What is the best practice for infection control measures for high-risk areas like the Hemodialysis Unit, and for patients with pre-existing kidney disease? |
| PICO 3: What precautions are needed for patients who are undergoing transplantation? This includes donors’ and recipients’ issues. |
| PICO 4: How to manage the children with suspected or confirmed to have COVID-19 and they are on IS? This includes patients with atypical hemolytic uremic syndrome (aHUS) on maintenance Eculizumab and potential drug interactions. |
| PICO 5: What are the special considerations for Low income countries that are applicable to all of the four previous health questions? |

# **Table S5 Future research recommendations**

| **Topic** | **Subtopic** | **Research Question** |
| --- | --- | --- |
| COVID-19 vaccination in children with kidney disease and immunocompromised or with co-morbidities | Selection of a specific COVID-19 vaccine | Compare effectiveness of different COVID-19 vaccines in children with kidney disease and immunocompromised or with co-morbidities |
|  |  | Compare safety and adverse effects of different COVID-19 vaccines in children with kidney disease and immunocompromised or with co-morbidities |
|  | COVID-19 antibody testing in routine clinical practice | Which antibody levels may be associated with protection against different variants of SARS-CoV-2? |
|  |  | Which antibody levels may be associated with protection against severe COVID-19 outcomes? |
|  | Role of hybrid immunity (natural SARS-CoV-2 infection plus vaccine) in protecting children with kidney diseases from severe COVID-19-related outcomes. | Is the “hybrid immunity state” associated with higher and more durable antibody responses compared with the non-hybrid state in order to recommend it as a formal public heath strategy to prevent severe outcomes among children with kidney diseases? |
