# Supplementary material for: IPNA clinical practice recommendations on care of pediatric patients with pre-existing kidney disease during seasonal outbreak of COVID-19
Source: Pediatr Nephrol. 2024 Dec 29;40(5):1795–815. doi: 10.1007/s00467-024-06565-5 (PMC11946955; doi:10.1007/s00467-024-06565-5)
Supplement: Supplementary file 2 — Supplementary file2 (DOCX 83 KB) [file 467_2024_6565_MOESM2_ESM.docx]

| **MAIN Q: Managing AND Pediatric Patients AND Pre-Existing Kidney Disease AND COVID-19 pandemic** | | | | | | | | | |
| --- | --- | --- | --- | --- | --- | --- | --- | --- | --- |
| **No#** | **Subgroup category** | **PICOTS** | | | | **Title (First Author, Year)** | **Design** | **Conclusion** | **Quality of Evidence** |
|  |  | **1** | **2** | **3** | **4** |  |  |  |  |
| 1 | nephrotic syndrome AND infection control AND heamodislysis |  | 2 | 3 |  | SARS-CoV-2 infection in children with chronic kidney disease (Krishnasamy, 2021) | multi center retrospective observational study | Children with CKD presenting with moderate-to-severe COVID-19 or in nephrotic syndrome relapse are at risk of severe complications, including severe AKI and mortality. | C |
| 2 |  | 1 |  |  |  | COVID-19 in children with chronic kidney disease: findings from the UK renal registry (Plumb, 2021) | letter | In light of these findings, alongside those from international colleagues,4 the BAPN has relaxed shielding criteria for children with kidney disease, details of which can be found on the Renal Association website (https://renal.org/covid-19/). This work, along with emerging evidence from other specialties, has enabled the Royal College of Paediatrics and Child Health to revise recommendations5 which have since been adopted by the UK government. | X |
| 3 |  | 1 |  |  |  | Outcomes and risk factors for death among hospitalized children and adolescents with kidney diseases and COVID-19: an analysis of a nationwide database (Vasconcelos, 2022) | retrospective analysis | Children and adolescents with KD had a higher risk of death compared with the non-KD cohort. The higher risk was associated with low oxygen saturation at admission, living in socioeconomically disadvantaged regions, and presence of other pre-existing comorbidities. A higher resolution version of the Graphical abstract is available as Supplementary information. | C |
| 5 | transplant |  |  |  | 4 | COVID-19 in pediatric kidney transplantation: a follow-up report of the Improving Renal Outcomes Collaborative (Varnell Jr, 2022) | follow up report | 2690 patients submitted data from 648 COVID-19 tests on 465 patients. Most patients required supportive care only and were treated as outpatients, 16% experienced inpatient care, and 5% experienced intensive care. Allograft complications were rare, with acute kidney injury most common (7%). There was 1 case of respiratory failure and 1 death attributed to COVID-19. Twelve centers that care for 1730 patients submitted complete testing data on 351 patients. The incidence of COVID-19 among patients at these centers was 4%, whereas the incidence among tested patients was 19%. Risk factors to predict a positive COVID-19 test included age > 12 years, symptoms consistent with COVID-19, and close contact with a confirmed case of COVID-19. | B |
| 6 | vaccine AND transplant |  | 2 |  | 4 | Considering a COVID-19 vaccine mandate for pediatric kidney transplant candidates (Wightman, 2022) | review | Finally, we will offer suggestions to be considered prior to the implementation of a COVID-19 vaccine mandate. | D |

| 7 |  | 1 |  |  |  | Heterogeneous Recommendations for School Attendance in Children With Chronic Kidney Diseases During the COVID-19 Pandemic in Europe (Schild, 2021) | cross sectional survey | COVID-19 causes educational deficits in the already vulnerable population of children with CKD. As the evidence for the course of COVID-19 in children with chronic diseases grows, rapidly adapted recommendations from pediatric societies could help reduce uncertainty among doctors, patients, and parents. | C |
| --- | --- | --- | --- | --- | --- | --- | --- | --- | --- |
| 8 | immunecompromised |  | 2 |  |  | Management of Canadian Pediatric Patients With Glomerular Diseases During the COVID-19 Pandemic: Recommendations From the Canadian Association of Pediatric Nephrologists COVID-19 Rapid Response Team (Robinson, 2020) | CPG | These guidelines are intended to promote optimal care delivery for children with existing or newly diagnosed glomerular diseases during the COVID-19 pandemic. The implications of modified care delivery, altered immunosuppression strategies, and limited access to existing resources remain uncertain. | A |
| 9 | infection control measures and heamodialysis |  |  | 3 |  | Canadian Association of Paediatric Nephrologists COVID-19 Rapid Response: Home and In-Center Dialysis Guidance (Alabbas, 2021) | CPG | We intend these recommendations to help provide the best care possible for pediatric patients prescribed in-center or home dialysis during the COVID-19 pandemic, a time of altered priorities and reduced resources. | A |
| 10 |  | 1 |  |  |  | Canadian Association of Paediatric Nephrologists COVID-19 Rapid Response: Guidelines for Management of Acute Kidney Injury in Children (Alabbas, 2021) | CPG | Given that most acute KRT related to COVID-19 is likely to be required in the pediatric intensive care unit initial setting, close collaboration and planning between critical care and pediatric nephrology programs are needed. Our group will update these suggestions with a supplement if necessary as newer evidence becomes available that may change or add to the recommendations provided. | A |
| 11 | immunecompromised AND transplant |  | 2 |  | 4 | Management of Pediatric Kidney Transplant Patients During the COVID-19 Pandemic: Guidance From the Canadian Society of Transplantation Pediatric Group (Teoh, 2020) | CPG | These recommendations are meant to serve as a guide to pediatric kidney transplant directors, clinicians, and administrators for providing the best patient care in the context of limited resources while protecting patients and health care providers wherever possible by limiting exposure to COVID-19. We recognize that recommendations may not be applicable to all provincial/local health authority practices and that they may not be delivered to all patients given the time and resource constraints affecting the individual provincial/local health jurisdiction. | A |

| 12 | immunecompromised AND nephrotic syndromeAND infection control AND heamodislysis AND transplant |  | 2 | 3 | 4 | Kidney Transplantation in a COVID-19-positive Pediatric Recipient (Hogan, 2021) | comment | This report suggests that the rapid improvement in our understanding of the disease and the immune response against it, together with the availability of rapid semiquantitative tests, may allow a more personalized approach to pretransplant screening especially in asymptomatic recipients. Our patient combined a high Ct value that has been previously associated with the absence of cultivable virus and the presence of anti-COVID-19 IgG suggesting a very low risk of posttransplant COVID-19-related complications. This type of personalized risk-assessment approach combining reverse transcription polymerase chain reaction results with the presence of specific IgG or T cells might be evaluated in children with other conditions requiring treatment. | X |
| --- | --- | --- | --- | --- | --- | --- | --- | --- | --- |
| 13 | transplant |  |  |  | 4 | Pediatric liver and kidney transplantation in the era of COVID-19: a follow-up study from a tertiary referral center in Iran  (Shafiekhani, 2021) | follow up report | the impact of COVID-19 pandemic on pediatric transplant outcomes and determine whether to continue pediatric transplant activity or not, and how policies intended our center has been effective in preventing COVID-19 among organ transplant recipients. | D |
| 14 | transplant |  |  |  | 4 | Impact of coronavirus disease-2019 on pediatric nephrology practice and education: an ESPN survey (Yazıcıoğlu, 2021) | survey | This survey showed a sharp decline in patient admissions and a significant decrease in kidney transplantation. Telemedicine and online teaching became essential tools, requiring integration into the current system. The prolonged and fluctuating course of the pandemic may pose additional challenges necessitating urgent and rational solutions. | C |
| 15 | immunecompromised AND transplant |  | 2 |  | 4 | Impact of COVID-19 Pandemic on Management of Pediatric Kidney Transplant Recipients (Saeed, 2021) | review | Reports to support evidence-based management of pediatric kidney transplant patients during the COVID-19 pandemic are lacking; therefore, expert opinion and available knowledge and experience remain subject to biases. | D |
| 16 | immunecompromised AND nephrotic syndrome AND transplant |  | 2 |  | 4 | Kidney implications of SARS-CoV2 infection in children (Bjornstad, 2022) | review | This review provides an update on our current understanding of SARS-CoV2 for pediatric nephrologists and highlights knowledge gaps to be addressed by future research during this ongoing pandemic, particularly the social disparities magnified during this period. | D |

| 17 |  | 1 |  |  |  | Acute kidney injury in critically Ill children and young adults with suspected SARS-CoV2 infection (Basu, 2021) | Sequential point-prevalence study | AKI and severe AKI occur commonly in critically ill children with SARS-CoV2 infection, more than double the historical standard. Further investigation is needed during this continuing pandemic to describe and refine the understanding of pediatric AKI epidemiology and outcomes. | C |
| --- | --- | --- | --- | --- | --- | --- | --- | --- | --- |
| 18 |  | 1 |  |  |  | Acute kidney injury in COVID-19 pediatric patients in North America: Analysis of the virtual pediatric systems data (Raina, 2022) | retrospective analysis | This is one of the first large scale studies to analyze AKI among pediatric COVID-19 patients admitted to the ICU in North America. Although the course of the COVID-19 virus appears milder in the pediatric population, renal complications may result, increasing the risk of disease complication and mortality. | B |
| 19 | vaccine AND nephrotic syndrome |  | 2 |  |  | Pathology findings in pediatric patients with COVID-19 and kidney dysfunction  (Nomura, 2022) | multi-institutional cohort | Although uncommon, COVID-19-associated kidney injury can have significant morbidity in the unvaccinated pediatric and adolescent population. A higher resolution version of the Graphical abstract is available as Supplementary information. | B |
| 20 | infection control AND heamodislysis |  |  | 3 |  | Acute Kidney Injury and Special Considerations during Renal Replacement Therapy in Children with Coronavirus Disease-19: Perspective from the Critical Care Nephrology Section of the European Society of Paediatric and Neonatal Intensive Care (Deep, 2021) | review | Intermittent hemodialysis can also be used in patients who are hemodynamically stable. The keys to successfully managing pediatric AKI in a pandemic are flexible use of resources, good understanding of dialysis techniques, and teamwork. | C |
| 21 |  | 1 |  |  |  | COVID-19: experiences of lockdown and support needs in children and young adults with kidney conditions (Tse, 2021) | pop based online survey | This survey demonstrates substantial concern and need for accurate tailored advice for CYA based on individualized risks to improve shared decision making. | A |
| 22 | transplant |  |  |  | 4 | This survey demonstrates substantial concern and need for accurate tailored advice for CYA based on individualised risks to improve shared decision making. (Gulati, 2021) | comment | Telenehrology offered an effective method for providing pediatric nephrology services during the period of pandemic lockdown, when access to paediatric nephrology care was limited. It is also an effective modality in providing individualised tailored advice to this vulnerable segment of the population. | X |
| 23 | immunecompromised AND nephrotic syndromeAND infection control AND heamodislysis |  | 2 | 3 |  | Impact of COVID-19 pandemic on use of rituximab among children with difficult nephrotic syndrome (Sinha, 2021) | questionnaire | despite the COVID-19 pandemic, rituximab continues to play an important role in the management of cDNS. In comparison to reports on the general paediatric population11 neither the risk of acquiring SARS-CoV-2 infection nor the risk of symptomatic disease and dismal outcomes appear to be increased among patients with cDNS receiving rituximab. | C |

| 24 |  | 1 |  |  |  | chrome-extension://efaidnbmnnnibpcajpcglclefindmkaj/https://ukkidney.org/sites/renal.org/files/BAPN/BAPN%20Covid%20advice%20for%20Children%20and%20Young%20People%20Sept%20%202021%20final.pdf (2022) | CPG |  | A |
| --- | --- | --- | --- | --- | --- | --- | --- | --- | --- |
| 25 | immunecompromised AND nephrotic syndromeAND infection control AND heamodislysis AND transplant |  | 2 | 3 | 4 | COVID-19 in children treated with immunosuppressive medication for kidney diseases (Marlais, 2021) | Cross-sectional study |  | A |
| 26 | immunecompromised |  | 2 |  |  | Impact of COVID-19 Pandemic in Children with CKD or Immunosuppression  (Mastrangelo, 2021) | letter | the incidence of COVID-19 is known to be low in the pediatric population, with a generally benign clinical trend. Considering the exposure to hospital environment in over a third of patients, our data show that even children in the advanced stages of CKD or on immunosuppressive therapy are at low risk of clinically relevant COVID-19. | X |
| 27 | immunecompromised |  | 2 |  |  | An analysis of chronic kidney disease as a prognostic factor in pediatric cases of COVID-19 (Dias Faria, 2021) | review | Immunosuppressant therapy has not been related with positive or negative effects in individuals with COVID-19, although current recommendations establish decreases in the dosage of some medications. To sum up with, CKD was not associated with more severe involvement in children diagnosed with COVID-19. Studies enrolling larger populations are still required. | D |
| 28 | AND infection control AND heamodislysis |  |  | 3 |  | COVID-19 Infection in Children and Young Adults with Chronic Kidney Disease: A Single Center Experience. (Swarnim, 2021) | retrospective study | The presenting features of COVID-19 were similar in children and young adults with underlying CKD compared to their healthy counterparts; however, the mortality rate and intensive care requirement were higher and duration of hospital stay too was longer. | C |
| 29 | infection control measures and heamodialysis |  |  | 3 |  | COVID-19 disease in Pakistani children with chronic kidney disease on hemodialysis – a tertiary care experience (Akhtar, 2022) | retrospective study | Our study showed that even children in the advanced stages of CKD were at low risk of developing COVID-19 disease. More than 50% of the patients were discharged uneventfully while 20% of the subjects expired due to severe COVID disease. | C |

| 30 | vaccine AND nephrotic syndromeAND infection control AND heamodislysis AND transplant |  | 2 | 3 | 4 | Intrinsic Kidney Pathology Following COVID-19 Infection inChildren and Adolescents: A Systematic Review (Henry H, 2022) | SR | This systematic review highlights the various intrinsicpathological kidney manifestations in children and adolescents as a result of acute COVID-19 infection.The clinical timeline and presentation of these cases support the mechanistic hypothesis betweenCOVID-19 infection and the onset of intrinsic kidney pathologies within this context. The progressiveintroduction of vaccination programs for children and adolescents may hopefully reduce the severityof COVID-19-associated illnesses, and pathological kidney manifestations in this population. | A |
| --- | --- | --- | --- | --- | --- | --- | --- | --- | --- |
| 31 | immunecompromised AND nephrotic syndromeAND infection control AND heamodislysis |  | 2 | 3 |  | COVID-19 in Children With Kidney Disease: A Report of 2 Cases (Basalely, 2021) | case report | The presentation of novel coronavirus disease 2019 (COVID-19) in children with kidney disease is largely unknown. We report on 2 children with kidney disease not receiving long-term immunosuppression who were hospitalized due to COVID-19. | D |
| 32 | vaccine AND immunecompromised AND nephrotic syndrome |  | 2 |  |  | COVID-19 and idiopathic nephrotic syndrome in children: systematic review of the literature and recommendations from a highly affected area (Morello, 2022) | SR | Children with INS, with or without immunosuppression, are not at higher risk of severe SARS-CoV-2 infection. Relapse is a possible complication, but steroid treatment is safe and effective. After summarizing the evidence, we have suggested recommendations for the management of children with INS during the pandemic and the vaccination campaign. | A |
| 33 |  | 1 |  |  |  | Renal Involvement in Pediatric Patients with COVID-19: An Up-to-date Review (Campos, 2021) | review | Further studies are necessary to establish risk factors for renal involvement in pediatric COVID-19 and to predict disease outcomes. | D |
| 34 |  | 1 |  |  |  | Acute kidney injury in paediatric inflammatory multisystem syndrome temporally associated with SARS-CoV-2 (PIMS-TS) is not associated with progression to chronic kidney disease (Stewart, 2022) | observational study | Despite a high incidence of AKI in PIMS-TS, renal recovery occurs rapidly with current therapies, and no patients developed chronic kidney disease. | B |
| 35 |  | 1 |  |  |  | Acute Kidney Injury in Pediatric Inflammatory Multisystem Syndrome Temporally Associated With Severe Acute Respiratory Syndrome Coronavirus-2 Pandemic: Experience From PICUs Across United Kingdom (Deep, 2020) | pop based online survey | Severe acute kidney injury occurred in just over a quarter of children admitted to United Kingdom PICUs with pediatric inflammatory multisystem syndrome temporally associated with severe acute respiratory syndrome coronavirus-2. Hyperferritinemia was significantly associated with severe acute kidney injury. Severe acute kidney injury was associated with increased duration of stay and ventilation. Although short-term outcomes for acute kidney injury in pediatric inflammatory multisystem syndrome temporally associated with severe acute respiratory syndrome coronavirus-2 appear good, long-term outcomes are unknown. | A |

| 36 |  | 1 |  |  |  | Kidney involvement in multisystem inflammatory syndrome in children: a pediatric nephrologist's perspective (Sethi, 2021) | review | The current review gives a pediatric nephrologist's perspective of the renal involvement in MIS-C, the incidence of AKI, the pathophysiology of AKI in MIS-C and the proposed therapeutic regimens available, including the need for kidney replacement therapy for a child with AKI associated with MIS-C. As the disease is rapidly evolving, more detailed clinical prospective studies are required to understand MIS-C and its role in AKI better. | D |
| --- | --- | --- | --- | --- | --- | --- | --- | --- | --- |
| 37 |  | 1 |  |  |  | Acute Kidney Injury in Pediatric Acute SARS-CoV-2 Infection and Multisystem Inflammatory Syndrome in Children (MIS-C): Is There a Difference? (Grewal, 2021) | retrospective chart review | Children with acute SARS-CoV-2 infection and MIS-C are at risk for AKI, with the risk being significantly greater with MIS-C. The pathogenesis of AKI in acute SARS-CoV-2 infection appears to be a combination of both renal hypo-perfusion and direct renal parenchymal damage whereas in MIS-C, the renal injury appears to be predominantly pre-renal from cardiac dysfunction and capillary leak from a hyperinflammatory state. These factors should be considered by clinicians caring for these children with a special focus on renal protective strategies to aid in recovery and prevent additional injury to this high-risk subgroup. | B |
| 38 | AND infection control AND heamodislysis |  |  | 3 |  | AKI in COVID-19-Associated Multisystem Inflammatory Syndrome in Children (MIS-C) (Lipton, 2021) | retrospective cohort study | Although children with MIS-C may develop AKI, our study suggests that most experience mild disease, swift resolution, and promising outcome. Older age, increased inflammation, and left ventricular systolic dysfunction may be risk factors. Our study highlights the substantial differences in epidemiology and outcomes between AKI associated with pediatric MIS-C versus primary COVID-19 infection. | B |
| 39 |  | 1 |  |  |  | Acute kidney injury in children with COVID-19: a retrospective study (Kari, 2021) | multicenter retrospective cohort study | AKI occurred in one-fifth of children with SARS-CoV-2 infection requiring hospital admission, with one-third of those requiring PICU. AKI was associated with increased morbidity and mortality, and residual renal impairment at time of discharge. | A |
| 40 |  | 1 |  |  |  | Critical analysis of acute kidney injury in pediatric COVID-19 patients in the intensive care unit (Raina, 2021) | comprehensive literature search | AKI has shown to be a negative prognostic factor in adult patients with COVID-19 and now also in the pediatric cohort with high incidence and mortality rates. Additionally, our findings show a strong comparison in epidemiology between adult and pediatric COVID-19 patients; however, they need to be confirmed with additional data and studies. | B |

| 41 |  | 1 |  |  |  | Acute kidney injury in pediatric patients hospitalized with acute COVID-19 and multisystem inflammatory syndrome in children associated with COVID-19 (Basalely, 2021) | retrospective study | Acute kidney injury in acute-COVID-19 and MIS-C may be related to inflammation and/or dehydration. Further research in larger pediatric cohorts is needed to better characterize risk factors for acute kidney injury in acute-COVID-19 and with MIS-C consequent to COVID-19. | B |
| --- | --- | --- | --- | --- | --- | --- | --- | --- | --- |
| 42 |  | 1 |  |  |  | COVID-19 and the multisystem inflammatory syndrome in children: how vulnerable are the kidneys? (Chadha, 2021) | letter | Basalely et al. characterize acute kidney injury in pediatric patients with acute COVID-19 and multisystem inflammatory syndrome. Despite the associated morbidity, this cohort provides evidence of kidney recovery in most affected children. | X |
| 43 |  | 1 |  |  |  | Can microalbuminuria be an ındicator of renal ınvolvement in pediatric Covid 19 patients? (Özlü, 2022) | prospectively evaluated 100 pediatric patients | Although there was no difference between the groups with different disease course; microalbuminuria is detected in an important ratio of pediatric patients with COVID 19 in this study. In the highlight of our findings we suggest that urinary findings of pediatric COVID patients should be carefully evaluated. | B |
| 44 | immunocompromised |  | 2 |  |  | Hematuria as an Early Sign of Multisystem Inflammatory Syndrome in Children: A Case Report of a Boy with Multiple Comorbidities and Review of Literature (Generalić, 2021) | case report | Despite numerous reports of MIS-C cases in children, there are still many uncertainties regarding the clinical presentation and laboratory findings, as well as mechanisms beyond this intriguing disorder. In our case, for the first time hematuria is reported as an early symptom of MIS-C. We strongly believe that reporting various manifestations and outcomes in MIS-C patients will lead to improved diagnosis, treatment, and overall understanding of this novel inflammatory condition. | D |
| 45 | immunecompromised AND nephrotic syndrome |  | 2 |  |  | Coronavirus Disease-2019 in Children with Primary Kidney Disease: A Case series (Meshram, 2021) | ces series | They all responded well to oral azithromycin and supportive management. None of them received chloroquine, corticosteroids, or monoclonal antibodies. All three recovered without complications. | D |
| 46 |  | 1 |  |  |  | COVID-19 Among Children with Chronic Renal Diseases in Qatar (CCCRDQ) (2022) | Brief summary | Prevalence of COVID-19 infection between patients with chronic kidney diseases, and the role of COVID-19 infection in increasing the relapses and deterioration of chronic kidney diseases. | X |
| 47 | vaccine |  | 2 |  |  | Vaccine Attitudes and COVID-19 Vaccine Intention Among Parents of Children with Kidney Disease or Primary Hypertension. (Wang, 2022) | Cross-sectional study | Their reasons varied but key issues included the need for information pertinent to their child and consistent message from doctors and other healthcare providers. These findings may inform an effective vaccine campaign to protect children with kidney disease and hypertension. | C |
| 48 |  | 1 |  |  |  | POS-033 Acute Kidney Injury in Children with COVID-19 (J. Kari, 2021) | abstract | AKI occurred in one-fifth of children with SARS-CoV-2 infection requiring hospital admission, with one-third of those requiring PICU. AKI was associated with increased morbidity and mortality, and residual renal impairment at time of discharge. | X |

| 49 |  | 1 |  |  |  | Renal Involvement in COVID-19 Among Iranian Children (Mohkam, 2021) | cross-sectional study | The prevalence of AKI was high in patients with COVID-19 infection hospitalized in our tertiary hospital. We also found that a decrease in renal function was associated with a higher risk of mortality. Overall, early detection of AKI and effective treatment may help reduce mortality in patients with COVID-19. | C |
| --- | --- | --- | --- | --- | --- | --- | --- | --- | --- |
| 50 |  | 1 |  |  |  | Guidance on COVID-19 for Pediatric Kidney Disease Patients (Greenbaum, 2020) | CPG |  | A |
| 51 | immunecompromised |  | 2 |  |  | Save the kidneys in COVID-19 (Philip P, 2020) | comment | it is vital that we create awareness to actively look out for AKI, diagnose and prevent AKI pro-actively and have contingency plans in place to initiate KRT in cases that fulfill the criteria to receive KRT. We thank the authors again for their valuable initiative in this rapidly changing global crisis. | X |
| 52 | infection control AND heamodislysis |  |  | 3 |  | Food Insecurity During COVID-19 in Children with End-Stage Kidney Disease: A Pilot Study (Chan, 2022) | pilot study | Food insecurity was common among children with ESKD on chronic dialysis during the COVID-19 pandemic. Children with food insecurity had a greater increase in their phosphorus levels during the pandemic than did food secure children. Further exploration into how food resources such as an onsite food pantry impacts food insecurity and phosphorus control in children with ESKD is essential. | D |
| 53 |  | 1 |  |  |  | Acute kidney injury among pediatric COVID-19 patients admitted to the ICU in North America (Sai, 2022) | retrospective study | The study observed a higher incidence of AKI among pediatric COVID-19 patients in ICUs than previously reported by multiple studies. Moreover, mortality rates were higher among patients in the AKI group.Given the retrospective nature of the study, a few limitations exist. Notably, the results presented here are representative of North America only from select pediatric ICUs in the region and could not be applied globally. Notwithstanding the lower risk of severe COVID-19 in children, it is critical to continue to build the knowledge base of COVID-19-related manifestations in children. A better understanding of the disease could potentially address the increased morbidity and mortality rates in the future. | C |
| 54 |  | 1 |  |  |  | Coronavirus (COVID-19) – information for children, young people and families from the Kidney team (2020) | pamphlet |  | X |

| 55 |  | 1 |  |  |  | AKI in Children Hospitalized with COVID-19 (Socha, 2021) | retrospective cohort study | the researchers said, “In the setting of COVID-19, AKI occurred in approximately one-fifth of our hospitalized children, and more than one-third of those required PICU admission. AKI is more commonly found in younger children and in those with comorbid conditions. AKI is associated with increased mortality and morbidity. A small proportion of children with AKI can develop residual renal impairment at the time of discharge. Nonetheless, it tends to be milder than in adults, with a lower incidence of oliguria and less need for RRT.” | C |
| --- | --- | --- | --- | --- | --- | --- | --- | --- | --- |
| 56 | immunecompromised |  | 2 |  |  | COVID-19: Multisystem inflammatory syndrome in children (MIS-C) clinical features, evaluation, and diagnosis (Son, 2022) | review | Different case definitions were used in different studies, which may explain some of the variability in the reported frequency of these findings. As more is learned about MIS-C, it is becoming apparent that there is a wide spectrum of disease severity (figure 1) (see 'Spectrum of disease' below). Initial smaller case series largely reported the most severe end of the spectrum, resulting in a high reported incidence of shock, myocardial involvement, and respiratory failure. It is possible that as recognition of milder forms of MIS-C increases, the incidence of shock, left ventricular (LV) dysfunction, respiratory failure, and acute kidney injury will be lower. | D |
| 57 |  | 1 |  |  |  | A narrative review of care for patients on maintenance kidney replacement therapy during the COVID-19 era (Zhao, 2021) | review | We concluded that the pandemic has challenged the current provision of care and has a profound influence on the convey of renal care. | D |
| 58 | vaccine |  | 2 |  |  | Vaccine Attitudes and COVID-19 Vaccine Intention Among Parents of Children with Kidney Disease or Primary Hypertension (Wang, 2022) | Sequential explanatory mixed-methods design; survey followed by in-depth interviews. | Two-thirds of parents of children with kidney disease or hypertension were unsure or unwilling to vaccinate their child against COVID-19. Higher hesitancy towards routine childhood and influenza vaccination was associated with hesitancy towards COVID-19 vaccines. Enhanced communication of vaccine information relevant to kidney patients in an accessible manner should be examined as a means to reduce vaccine hesitancy. | C |
| 59 |  | 1 |  |  |  | COVID-19 - guidance for management of children admitted to hospital and for treatment of non-hospitalized children at risk of severe disease (Whittaker, 2022) | review | Paracetamol is the first line antipyretic. Ibuprofen should be avoided in children with poor fluid intake or suspected AKI, but this is related to the risk of kidney damage rather than worsening COVID-19. RCPCH has recommended that parents treat symptoms of fever or pain related to COVID-19 with either paracetamol or ibuprofen. | D |

| 60 |  | 1 |  |  |  | SARS-CoV-2 infection increases risk of acute kidney injury in a bimodal age distribution (Bjornstad, 2022) | Secondary analysis of ongoing prospective international cohort registry | SARS-CoV2-related AKI is common with a bimodal age distribution that is not fully explained by known risk factors or confounders. As the pandemic turns to disproportionately impacting younger individuals, this deserves further investigation as the presence of AKI and SARS-CoV2 infection increases hospital mortality risk. | C |
| --- | --- | --- | --- | --- | --- | --- | --- | --- | --- |
| 61 |  | 1 |  |  |  | Managing Children With Renal Diseases During the COVID-19 Pandemic (Vasudevan, 2020) | CPG | The present guidelines of the Indian Society of Pediatric Nephrology on managing patients with kidney diseases during the COVID-19 pandemic are based on current literature and expert views. While children constitute a small proportion of patients with COVID-19, those with chronic disorders constitute a high-risk group and at-risk for adverse outcomes. Therapeutic guidelines are likely to change as evidence emerges from large case series and randomized controlled trials. | A |
| 62 |  | 1 |  |  |  | Recommendations Of The Brazilian Society Of Nephrology Regarding Pediatric Patients On Renal Replacement Therapy During The Covid-19 Pandemic (Tavares, 2020) | CPG | Despite initial observations of higher mortality rates in specific age groups (the elderly) and with comorbidities (obese, diabetics, and those with cardiovascular diseases), patients with chronic kidney disease (CKD) on RRT are particularly prone to develop COVID-19. Specific measures must be taken to reduce the risk of contracting SARS-CoV-2 and developing COVID-19, especially during transport to dialysis facilities, as well as on arrival and in contact with other patients. | A |
| 63 |  | 1 |  |  |  | Rapid response in the COVID-19 pandemic: a Delphi study from the European Pediatric Dialysis Working Group (Eibensteiner, 2020) | CPG | As limited quantitative evidence is available in real time during the rapid spread of the COVID-19 pandemic, qualitative expert knowledge and experience represent the best evidence available. This Delphi exercise demonstrates that use of mixed methodologies embedded in an established network of experts allowed prompt analysis of pediatric nephrologists' response to COVID-19 during this fast-emerging public health crisis. Such rapid sharing of knowledge and local practices is essential to timely and optimal guidance for medical management of specific patient groups in multi-country health care systems such as those of Europe and the US. | A |
| 64 | immunecompromised |  | 2 |  |  | Coronavirus disease 2019 (COVID-19) in two pediatric patients with kidney disease on chronic immunosuppression: A case series (Rawson, 2021) | case report | This report describes the mild clinical disease course of COVID-19 in two pediatric patients with chronic kidney disease, one on hemodialysis and both on chronic immunosuppression. We review treatment in these patients, as well as our measures to reduce transmission among our hemodialysis patients and staff. | D |

| 65 |  | 1 |  |  |  | Effect of adult COVID-19 surge on the provision of kidney replacement therapy in children (Deep, 2020) | comment | The most important weapons are collaborative team work, timely dissemination of knowledge by education and training, developing resilience in the system and being innovative and flexible in the best interests of the patient. These are unprecedented times; the spectrum of clinical presentation of children affected by COVID-19 is evolving, and we, as clinicians, will need to adapt to this new “unknown”. | D |
| --- | --- | --- | --- | --- | --- | --- | --- | --- | --- |
| 66 |  | 1 |  |  |  | Psychological Concerns of Children Undergoing Kidney Transplantation During the Pandemic: Single-centre Experience (Paessler, 2021) | retrospective analysis | While detailed counselling and additional safety precautions contributed to a good experience, patients and parents still demonstrated fear towards transplantation. | C |
| 67 | immunecompromised |  | 2 |  |  | Arterial abnormalities identified in kidneys transplanted into children during the COVID-19 pandemic (Berteloot, 2021) | observational study | Because the diagnosis of COVID-19 is challenging in children, we recommend pretransplant monitoring of graft recipients and their parents by monthly RT-PCR and serology. We suggest balancing the risk of postviral graft vasculitis against the risk of prolonged dialysis when considering transplantation in a child during the pandemic. | B |
| 68 |  | 1 |  |  |  | Rhabdomyolysis and Acute Renal Failure in an Adolescent With Coronavirus Disease 2019 (Samies, 2020) | case report | Here, we report an adolescent with COVID-19-associated rhabdomyolysis who required hemodialysis due to acute kidney injury. Pediatric providers should consider rhabdomyolysis and the possibility of acute renal failure in children with COVID-19. | D |
| 69 |  | 1 |  |  |  | Role of pediatric nephrologists in managing adults with AKI due to COVID-19 (Lipton, 2020) | comment | While other institutions accepted adults into pediatric units or redeployed pediatric providers to adult units, our division used a mixed model in mobilization necessitated by the significant need. Through this process, we have also learned that cross-training of physicians and nurses in caring for both adults and children should be integrated into better preparedness for public health emergencies should they arise again. | X |
| 70 | immunecompromised |  | 2 |  |  | Managing Children with Renal Diseases during COVID-19 Pandemic (Vasudevan, 2020) | review | Our preparedness for managing this vulnerable group of children is the need of the hour. The purpose of this article is to provide guidance to caregivers and health care personnel involved in management of children with renal diseases and to ensure patient well-being, while protecting staff from infection. | D |
| 71 | immunecompromised |  | 2 |  |  | COVID-19 impact poses low risk to children with advanced CKD, immunosuppression (Melissa J, 2021) | retrospective analysis | These findings demonstrate that even children who would appear to be at high-risk (based on adult cohorts with similar characteristics), also have a low risk of “clinically relevant COVID-19.” | D |

| 72 |  | 1 |  |  |  | Renal involvement in children with COVID-19 infection (Momtaz, 2020) | mini review | This mini-review attempts to consider the publications focused on the COVID-19 infection among children with emphasis on renal involvement and the treatment approach of this complication. | D |
| --- | --- | --- | --- | --- | --- | --- | --- | --- | --- |
| 73 |  | 1 |  |  |  | The Impact of the COVID-19 Outbreak on the Medical Treatment of Chinese Children with Chronic Kidney Disease (CKD) : A Multicenter Cross-section Study in the Context of a Public Health Emergency of International Concern (Zhang, 2020) | anonymous online questionnaire survey | The COVID-19 outbreak has affected the medical treatment of children with CKD. Online consultation, medication delivery and psychological counselling are the greatest needs reported by patients and their families and could especially provide solutions for the management of low income children with CKD in remote rural areas in the context of the COVID-19 epidemic. | B |
| 74 | immunecompromised |  | 2 |  |  | Consensus recommendations for the care of children receiving chronic dialysis in association with the COVID-19 epidemic (Shen, 2020) | CPG | Children on chronic dialysis are particularly susceptible to COVID-19. In order to effectively prevent and control the transmission of SARS-CoV-2 among children who receive maintenance dialysis, we formulated this set of recommendations based on infectious disease guidelines and our experience with the COVID-19 epidemic, which healthcare staff in pediatric dialysis centers can refer to. We suspect that these recommendations may well apply during epidemics related to other respiratory viruses as well. These recommendations will be updated as new information regarding SARS-CoV-2 and COVID-19 becomes available. | A |
| 75 | vaccine immunecompromised |  | 2 |  |  | Perspective on COVID-19 vaccination in patients with immune-mediated kidney diseases: consensus statements from the ERA-IWG and EUVAS (Stevens, 2022) | corrected proof | The Immunonephrology Working Group and European Vasculitis Society recommend that patients with immune-mediated kidney diseases follow national guidance on vaccination. Booster doses based on antibody measurements could be considered. | C |
| 76 | vaccine |  | 2 |  |  | Be aware of acute kidney injury in critically ill children with COVID-19 (Wang, 2020) | retrospective observational study | Critically ill children with COVID-19 may develop AKI, especially following prodromal gastrointestinal symptoms. An inflammatory storm and complement-mediated injury may underlie AKI development in children with COVID-19. Our study supports implantation of PE and CKRT in management of critically ill patients with AKI. | B |
| 77 |  | 1 |  |  |  | COVID-19 in Children: Clinical Approach and Management (Sankar, 2020) | review | An early intubation is preferred over non-invasive ventilation or heated, humidified, high flow nasal cannula oxygen, as these may generate aerosols increasing the risk of infection in health care personnel. To prevent post discharge dissemination of infection, home isolation for 1–2 wk may be advised. As of now, no vaccine or specific chemotherapeutic agents are approved for children. | D |

| 78 |  | 1 |  |  |  | Kidney Replacement Therapy in COVID-19 Induced Kidney Failure and Septic Shock: A Pediatric Continuous Renal Replacement Therapy [PCRRT] Position on Emergency Preparedness with Resource Allocation  (Raina, 2020) | HYPOTHESIS AND THEORY article | COVID-19 is another emerging respiratory virus that has severely challenged the health care system around the world. However, the adult cases have been reported to be more prevalent and severe in comparison to pediatric cases.Thus, in this position paper, we present an emergency preparedness plan with resource allocation if conditions in the pediatric population worsened dramatically. There is a limitation as this is a position paper grounded on theory of the pathogenesis and anecdotal publication by Ronco et al. (26) and based primarily on adult data and the limited studies available on COVID-19. However, there currently are no effective treatments available and therefore, we suggest the use of high volume CVVHDF in critically ill pediatric COVID-19 patients in the setting of sepsis and MODS. If CVVHDF or the resources required are not available, other KRT modalities, such as CVVHD, SLEDD-f and PD can be utilized. Additionally, incorporation of ECMO circuit with the CVVHDF machinery may improve overall outcomes in COVID-19 patients requiring ventilatory support. | X |
| --- | --- | --- | --- | --- | --- | --- | --- | --- | --- |
| 79 |  | 1 |  |  |  | Social Support and Loneliness Among Chinese Caregivers of Children with Chronic Kidney Disease During the COVID-19 Pandemic: A Propensity Score Matching Analysis (Shi, 2021) | cross-sectional study | we found that caregivers of children with CKD had poorer social support and greater loneliness than caregivers of healthy children during the COVID-19 pandemic. We also found that social support was inversely correlated with loneliness. Potential interventions can be developed and implemented to support these caregivers financially and psychologically during the current pandemic and future pandemics to improve the quality of life of the caregivers and their children. | C |
| 80 | immunecompromised, nephrotic syn, transplant |  | 2 |  | 4 | De novo collapsing glomerulopathy in a pediatric kidney transplant recipient with COVID-19 infection (Levenson, 2021) | case report | Pediatric kidney transplant recipients can develop severe COVID-19-related kidney complications. Judicious immunosuppression modulation is necessary to balance infection and rejection risk. | D |
| 81 |  | 1 |  |  |  | Distress, anxiety, and its correlates among caregivers of children with kidney diseases during COVID-19 pandemic lockdown (Sharmaa, 2022) | online survey | A high prevalence of stress, anxiety, and depression along with insomnia was detected among the caregivers of children with kidney diseases during the COVID-19 pandemic. | C |
| 82 |  | 1 |  |  |  | Favipiravir use in children with COVID-19 and acute kidney injury: is it safe? (Ozsurekci, 2021) | trial | Favipiravir seems a suitable therapeutic option in patients affected by COVID-19 with kidney injury without a need for dose adjustment. | B |

| 83 |  | 1 |  |  |  | Providing Medical Services Online to Children With Chronic Kidney Disease During the COVID-19 Pandemic (Menon, 2021) | letter | Expensive or less easily procured drugs were made available using government schemes. This was not easy for children staying in other districts who needed to travel long distances to reach the hospital. In such cases, liaison was established with the Reproductive and Child Health (RCH) officer of those districts, or the doctors in peripheral rural hospitals, who went out of their way to make the drugs available locally. | X |
| --- | --- | --- | --- | --- | --- | --- | --- | --- | --- |
| 84 | heamodialysis and transplantation |  |  | 3 | 4 | COVID-19 in pediatric patients undergoing chronic dialysis and kidney transplantation (Canpolat, 2021) | multicenter observational study | While most cases are asymptomatic or have a mild disease course, pediatric patients undergoing dialysis and a kidney transplant are at increased risk for COVID-19. | B |
| 85 | immunecompromised |  | 2 |  |  | COVID-19 in children and young adults with kidney disease: risk factors, clinical features and serological response (Weinbrand-Goichberg, 2021) | Study population | Unlike COVID-19 in adult patients with kidney disease, in our cohort of children and young adults, COVID-19 incidence was similar to the general population and all cases were mild. It may be unnecessary to impose severe restrictions on this patient population during the pandemic. | C |
| 86 | nephrotic |  | 2 |  |  | Clinical Profile and Outcome of COVID -19 in Children with Pre-Existing Renal Disease (Khondaker, 2021) | retrospective study | Covid -19 is frequent in patients with a history of kidney diseases and it may present with an atypical presentation like hypertension and or hematuria. Hence, clinicians should increase their awareness and concern to deal with COVID-19 infection among renal patients. | D |
| 87 | nephrotic |  | 2 |  |  | New-Onset Nephrotic Syndrome in a Child Associated With COVID-19 Infection  (Shah, 2020) | case report | COVID-19 can be associated with new-onset nephrotic syndrome in children. The patient responded well to the standard-dose prednisone treatment that is typically used for new-onset nephrotic syndrome. | D |
| 88 | immunecompromised, nephrotic syn, transplant |  | 2 |  | 4 | SARS-CoV-2 infection in Spanish children with chronic kidney pathologies (Melgosa, 2020) | Brief report | COVID-19 disease appears to have a similar clinical course in children with underlying chronic renal pathologies, even in immunosuppressed cases, as in healthy children of the same age; however, special attention must be paid to fluid management and drug dose adjustment. | D |
| 89 |  | 1 |  |  |  | Acute necrotizing glomerulonephritis associated with COVID-19 infection: report of two pediatric cases (Basiratnia, 2021) | case report | Both reported cases had an acute presentation of kidney injury with positive nasopharyngeal PCR test for COVID-19. Based on the data review by the researchers, this is the first report of acute necrotizing GN associated with COVID-19 infection. | D |
| 90 | immunecompromised, transplant |  | 2 |  | 4 | Impact of COVID-19 Pandemic on Management of Pediatric Kidney Transplant Recipients (Saeed, 2021) | review | There is a paucity of literature to support evidencebased management of PKT patients during the COVID-19 pandemic. The expert opinions and available knowledge and experiences that are available are subject to biases associated with this level of evidence. | C |
| 91 |  | 1 |  |  |  | Blood filters in children with COVID-19 and acute kidney injury: A review (Raina, 2022) | review | Therapeutic effects of these blood filters range from cytokine removal (CytoSorb, HA330, HCO/MCO), endotoxin removal (Toraymyxin, CPFA), both cytokine and endotoxin removal (oXiris), and nonspecific removal of proteins (PMMA) that have already been established and can be used to mitigate the various effects of the cytokine storm syndrome in COVID-19. | D |
| 92 | transplant |  |  |  | 4 | Effects of COVID-19 pandemic on pediatric kidney transplant in the United States (Charnaya, 2020) | SRTR data | The COVID-19 pandemic initially reduced access to kidney transplantation among pediatric patients in the USA but has not had a sustained effect. | C |
| 93 | immunecompromised |  | 2 |  |  | Is coronavirus pandemic-related anxiety higher in children with chronic kidney disease than healthy children? (Erfidan, 2021) | cross-sectional, case control | The current COVID-19 pandemic is a disaster that children encounter for the first time in their lives. It does not exclusively cause anxiety among children with chronic kidney diseases but also affects healthy children. | C |
| 94 | infection control |  | 2 | 3 |  | First Reported Nosocomial Outbreak of Severe Acute Respiratory Syndrome Coronavirus 2 in a Pediatric Dialysis Unit (Schwierzeck, 2021) | single-center experience | Person-to-person transmission was at the heart of a hospital outbreak of SARS-CoV-2 between healthcare workers (HCWs) and patients in the pediatric dialysis unit at UHM. Semiquantitative rRT-PCR results suggest that individuals with high viral load pose a risk to spread SARS-CoV-2 in the hospital setting. Our epidemiological observation highlights the need to develop strategies to trace and monitor SARS-CoV-2–infected HCWs to prevent COVID-19 outbreaks in the hospital setting. | C |
| 95 |  | 1 |  |  |  | Efficacy of remdesivir for hospitalized COVID-19 patients with end stage renal disease (Selvaraj, 2021) | retrospective, multicenter study | Larger studies are justified to study the effects of remdesivir in this high-risk population with end stage kidney disease on dialysis. | B |
| 96 |  | 1 |  |  |  | Acute Kidney Injury in Multisystem Inflammatory Syndrome in Children (MIS-C): a Case Report (Lee, 2020) | case report | Pediatric providers, during a pandemic with imperfect testing, must be keenly aware of how varied the pathogenesis of COVID-19 can be in children. | D |
| 97 |  | 1 |  |  |  | Acute Kidney Injury and Special Considerations during Renal Replacement Therapy in Children with Coronavirus Disease-19: Perspective from the Critical Care Nephrology Section of the European Society of Paediatric and Neonatal Intensive Care (Deep A, 2021) | review | Increased doses of unfractionated heparin, combination of heparin and regional citrate anticoagulation, or combination of prostacyclin and heparin might be used. If infusion pumps to deliver anticoagulants are limited, the administration of low-molecular-weight heparin might be considered. Alternatively in children, acute peritoneal dialysis can successfully control both fluid and metabolic disturbances. Intermittent hemodialysis can also be used in patients who are hemodynamically stable. The keys to successfully managing pediatric AKI in a pandemic are flexible use of resources, good understanding of dialysis techniques, and teamwork. | D |

| 98 | immunecompromised |  | 2 |  |  | Renal dysfunction in hospitalised children with COVID-19 (Stewart, 2020) | correspondance | Our data highlight the importance of renal function surveillance in all hospitalised paediatric cases of COVID-19, while simultaneously avoiding factors that exacerbate kidney injury, such as hypovolaemia and the use of nephrotoxic drugs. Standard care should involve screening for nephritis and follow-up for long-term sequelae of acute kidney injury, such as hypertension and proteinuria. A collaborative multicentre approach should be sought to quantify rates of renal dysfunction in paediatric cases of COVID-19. Further research should seek to compare SARS-CoV-2-positive paediatric patients against controls with other infectious causes of hypovolaemic or hyperinflammatory shock to evaluate whether COVID-19 predisposes children and adolescents to a disproportionately higher risk of acute kidney injury. | X |
| --- | --- | --- | --- | --- | --- | --- | --- | --- | --- |
| 99 | immunecompromised, nephrotic syn, transplant |  | 2 |  | 4 | IPNA clinical practice recommendations for the diagnosis and management of children with steroid-resistant nephrotic syndrome (Trautmann, 2020) | CPG | The team performed a systematic literature review on 9 clinically relevant PICO (Patient or Population covered, Intervention, Comparator, Outcome) questions, formulated recommendations and formally graded them at a consensus meeting, with input from patient representatives and a dietician acting as external advisors and a voting panel of pediatric nephrologists. Research recommendations are also given. | A |
| 100 | immunecompromised, transplant |  | 2 |  | 4 | Effect of COVID-19 on Kidney Disease Incidence andManagement (McAdams, 2021) | review | The chance of transmission puts in-center chronichemodialysis and other immunosuppressed patients at particularly increased risk. Limited data show that patientswith CKD are also at increased risk for more severe disease, if infected. Little is known about the virus’s effects onimmunocompromised patients with glomerular diseases and kidney transplants, which introduces challenges formanagement of immunosuppressant regimens. Although there are no standardized guidelines regarding themanagement of immunosuppression, several groups recommend stopping the antimetabolite in hospitalizedtransplant patients and continuing a reduced dose of calcineurin inhibitors. This comprehensive review criticallyappraises the best available evidence regarding the effect of COVID-19 on the incidence and management ofkidney diseases. Where evidence is lacking, current expert opinion and clinical guidelines are reviewed, andknowledge gaps worth investigation are identified. | D |

| 101 |  | 1 |  |  |  | SARS-CoV-2and pediatric solid organ transplantation: Currentknowns and unknowns (L’Huillier, 2021) | case report | Currently available data are still lacking in the pediatric SOT population, but data haveemerged in both the adult SOT and general pediatric population regarding the approachto COVID-19.The document provides expert opinion regarding prevention,diagnosis, and management of SARS-CoV-2infection among pediatric SOT candidatesand recipients. | D |
| --- | --- | --- | --- | --- | --- | --- | --- | --- | --- |
| 102 | Adult infection control |  |  | 3 |  | COVID-19 Prevention and Control in Dialysis Centers during the Pandemic: A Single-Center Experience (Gan, 2022) | single-center experience | This article provides key points in coping with COVID-19 in dialysis centers during this pandemic based on experience: (1) enforcing infection control management of dialysis centers, (2) training of hemodialysis patients and medical staffs, (3) screening for COVID-19 among patients and medical staffs, and (4) providing graded isolated dialysis to close contacts, suspected cases, and confirmed cases of COVID-19. We hope our single-center experience can be referenced by other dialysis centers around the world in coping with the COVID-19 pandemic. | C |
| 103 | Adult infection control |  |  | 3 |  | COVID-19 in dialysis units: A comprehensive review (Nogueira, 2021) | comprehensive review | In addition to preventive measures, this article briefly describes actions directed towards management of an outbreak of the severe acute respiratory syndrome coronavirus 2 (SARS-CoV-2) within a dialysis facility, the management of complications in dialysis patients with COVID-19 and overall data regarding the management of children with kidney disease. | C |
| 104 | Adult infection control |  |  | 3 |  | Infection Control Precautions and Care Delivery in Hemodialysis Unit during Coronavirus Disease 2019 Outbreak: A Case Series (Shou-Ci Hu, 2021) | A Case Series | Our study described a practical workflow for patient-centered management during COVID-19 outbreak. Potential risk factors and underlying clinical patterns were reported. Further studies regarding the efficacy of infection control precautions and practice protocols tailored for dialysis settings are warranted. | D |
| 105 | Adult infection control |  |  | 3 |  | Infection prevention measures for patients undergoing hemodialysis during the COVID-19 pandemic in Japan: a nationwide questionnaire survey (Sugawara, 2021) | a nationwide questionnaire survey | This survey revealed that most hemodialysis facilities in Japan had improved implementation of infection control measures and had shortage of PPEs and disinfectants, though some facilities did not implement infection prevention measures adequately, mainly due to the limited space of the facility. It may be recommended that each facility immediately establishes isolation measures to prepare for the pandemic of COVID-19. | A |
| 106 | Adult infection control |  |  | 3 |  | Risk of COVID-19 Disease, Dialysis Unit Attributes, and Infection Control Strategy among London In-Center Hemodialysis Patients (Caplin, 2021) | single-center experience | Rates of COVID-19 in the in-center hemodialysis population relate to individual factors, underlying community transmission, unit size, and layout. | C |

| 107 | Adult infection control |  |  | 3 |  | The keys to control a COVID-19 outbreak in a haemodialysis unit (Rincón, 2020) | single-center experience | Detection of asymptomatic SARS-CoV-2-positive patients is probably one of the key points to controlling an outbreak in an HD unit. Sharing health-care transportation to the dialysis unit, living in a nursing home and having been admitted to the reference hospital within the previous 2 weeks, are major risk factors for SARS-CoV-2 infection. | C |
| --- | --- | --- | --- | --- | --- | --- | --- | --- | --- |
| 108 | Adult infection control |  |  | 3 |  | COVID-19 and Dialysis Units: What Do We Know Now and What Should We Do? (Ikizler, 2020) | editorial | multiple organizations have developed guidance documents for the prevention and control of COVID-19 infection in the outpatient hemodialysis setting. Official guidance from the Centers for Disease Control and Prevention (CDC), Interim Guidance for Infection Prevention and Control Recommendations for Patients With Suspected or Confirmed COVID-19 in Outpatient Hemodialysis Facilities, can be freely accessed at the CDC6 and American Society of Nephrology7 websites. In addition, several dialysis organizations have developed their own guidance documents that are distributed to physicians who are related to these entities. Importantly, these documents are considered “interim” and are expected to evolve as more information is gathered. | X |
| 109 | Adult infection control |  |  | 3 |  | COVID-19 Outbreak in an Urban Hemodialysis Unit (Yau, 2020) | Retrospective cohort study. | Universal SARS-CoV-2 testing and universal droplet and contact precautions in the setting of an outbreak appeared to be effective in preventing further transmission. | B |
| 110 | Adult infection control |  |  | 3 |  | Infection control measures to prevent outbreaks of COVID-19 in Quebec hemodialysis units: a cross-sectional survey (Beaubien-Souligny, 2021) | a cross-sectional survey | Rates of COVID-19 infection among hemodialysis recipients in Quebec were elevated compared to the general population during the first year of the pandemic, and although hemodialysis units throughout the province implemented appropriate IPAC measures rapidly in the spring of 2020, many units were crowded and could not maintain physical distancing. Future hemodialysis units should be designed to minimize airborne and droplet transmission of infection. | c |
| 111 | Adult infection control |  |  | 3 |  | How we mitigated and contained the COVID-19 outbreak in a hemodialysis center: Lessons and experience (Ke-Su, 2020) | editorial | When hemodialysis patients with COVID-19 recovered, they were transferred to a quarantine ward for recovered patients for 14 days of observation. After 2 negative nucleic acid tests, the patient could be transferred to the uncontaminated hemodialysis center. If any healthcare personnel were confirmed with COVID-19 or had a probable case, they were also quarantined | X |

| 112 | Adult infection control |  |  | 3 |  | Core principles for infection prevention in hemodialysis centers during the COVID-19 pandemic (Chen, 2020) | editorial | we promote work–life balance for staff and encourage patients to take the initiative to participate. Our hemodialysis center has strived to achieve zero infection during the ongoing COVID-19 outbreak. | X |
| --- | --- | --- | --- | --- | --- | --- | --- | --- | --- |
| 113 | Adult infection control |  |  | 3 |  | Epidemiology of COVID-19 in an Urban Dialysis Center (Corbett, 2020) | single-center experience | The COVID-19 epidemic affected a large proportion of patients at this dialysis center, creating service pressures exacerbated by nursing staff illness. Details of the control strategy and characteristics of this epidemic may be useful for dialysis providers and other institutions providing patient care. | C |
| 114 | Adult infection control |  |  | 3 |  | Presentation and Outcomes of Patients with ESKD and COVID-19 (Valeri, 2020) | single-center experience | The association of COVID-19 with high mortality in patients with ESKD on dialysis reinforces the need to take appropriate infection control measures to prevent COVID-19 spread in this vulnerable population. | C |
| 115 | Adult infection control |  |  | 3 |  | Delivering Dialysis During the COVID-19 Outbreak: Strategies and Outcomes  (Roper, 2020) | single-center experience | Although these interventions are shown to have been effective in the initial phase of this pandemic, it will be important to maintain them in subsequent phases of increased SARS-Cov-2 transmission, such as may occur when social distancing strategies for the general population are relaxed. | C |
| **Footnote:**  PICO 1 Children/Adol COVID Kidney disease  PICO 2 Vaccine Immunecompromised Nephrotic Syndrome  PICO 3 infection control heamodyalisis  PICO 4 Transplantation Heamolytic uremic syn Low-income countries  Quality of Evidence  According to IPNA criteria in PPt.  Level A  Level B  Level C  Level D  Level X | | | | | | | | | |
